# Roadmap collaborative research on structured magnetic elastomers and their material behavior–bridging from fundamental and materials science to educational science

**DOI:** 10.1140/epje/s10189-026-00635-2

**Published:** 2026-09-25

**Authors:** Henning Reinken, Karl A. Kalina, Deniz C. Senel, Heinrich T. Roth, Nils Magin, Konstantin Gerstenberger, Lukas Fischer, Konstantin Zisiadis, Markus Heiber, Reza Azizmalayeri, Stefan Michel, Maximilian Lange, Nelly Pappermann, Muhammed Muhsin Abdul Azeez, Markus Kästner, Stefan Odenbach, Bianca Watzka, Günter K. Auernhammer, Andreas M. Menzel

**Affiliations:** 1 Otto-von-Guericke-Universität Magdeburg, Institut für Physik, Universitätsplatz 2, 39106 Magdeburg, Germany; 2 Institut für Festkörpermechanik, TU Dresden, George-Bähr-Straße 3c, 01069 Dresden, Germany; 3 I. Physikalisches Institut (IA), RWTH Aachen University, Sommerfeldstraße 16, 52074 Aachen, Germany; 4 Institut für Mechatronischen Maschinenbau, TU Dresden, Strehlener Straße 22–24, 01069 Dresden, Germany; 5 Leibniz-Institut für Polymerforschung Dresden e. V., Hohe Straße 6, 01069 Dresden, Germany

## Abstract

**Abstract:**

Magnetic elastomers, like magnetic fluids, consist of magnetizable particles in a carrier medium. In contrast to magnetic fluids, the carrier matrix is a soft elastic solid. Consequently, the particles are permanently held in place. Such positional fixation allows to use the structure of the spatial particle arrangement as a central degree of freedom to enhance the properties of the materials. Specifically, structural optimization can improve overall magnetostrictive and magnetorheological properties. Key questions concern the identification of optimized structures, their consequences on the overall material properties, and ways of transferring them into reality. Furthermore, to close the gap between pure fundamental and materials science on the one hand and education on the other hand, the contents are introduced into educational science and research. They provide a solid basis for context- and authenticity-based learning as well as related scientific investigations. We overview multiple challenges on this path, potential ways of solution, and we estimate further developments in the field. Clearly, this kind of challenge can only be addressed in an interdisciplinary and collaborative approach.

**Graphical abstract:**

**Figure:**
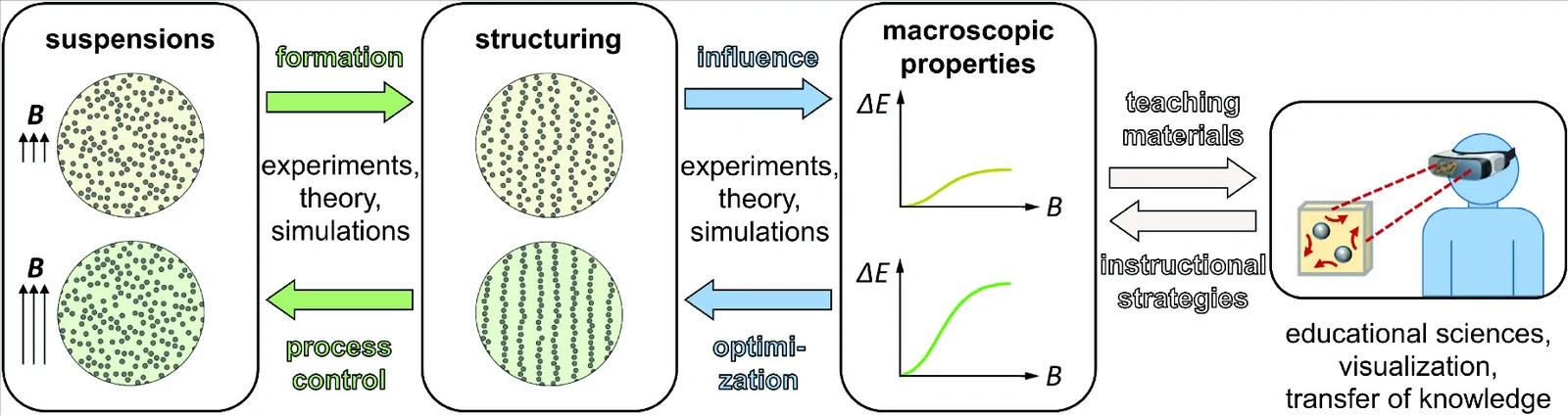

## Overview

Andreas M. Menzel, *Otto-von-Guericke-Universität Magdeburg, Magdeburg, Germany*

Magnetic hybrid materials, composed of discrete magnetic or magnetizable particles in a continuous carrier medium, have a long-standing history. Initially, research concentrated mostly on liquids containing the immersed particles [1–4]. Frequently, the resulting suspensions are referred to as ferrofluids when based on particles of nanometer dimensions, and as magnetorheological fluids for particles of micrometer sizes. Research extended to magnetic or magnetizable particles embedded in soft elastic carrier media, mostly of polymeric origin [5–8]. Following an analogous wording, the term ferrogels refers to materials containing particles of nanometer sizes, while magnetorheological elastomers contain particles on the micrometer scale. We will not focus on a historic recapitulation of the evolution of the field. Instead, we will define the systems under our current consideration, elucidate their potentials, and explain conceivable tracks that current and future research in the field may take. We do, however, mention that nature has created and developed corresponding systems already quite a long time ago. Examples are magnetosomes in magnetotactic bacteria [9] or small magnetic particles in the beaks of certain birds [10]. Both types of composite structures can support the regulation of the orientation of motion of these living creatures.

In our context, we focus on magnetorheological gels and elastomers as well as related materials and systems. Particle sizes range from a few up to several hundred micrometers in diameter [11, 12]. Such particles are too large to move through the polymeric mesh and are therefore trapped. They are embedded in elastomeric carrier media of elastic shear and Young moduli from a few Pa up to the scale of about 100 kPa [13, 14]. For brevity, we hereafter refer to these materials conventionally as “magnetic elastomers”.

There are, in principle, two major branches when considering the nature of the magnetic properties of the particles. We term them “magnetically soft”, if they show hardly any, ideally no magnetic remanence. Thus, their magnetization vanishes in the absence of any external magnetic field. Conversely, we term them “magnetically hard”, if they feature notable remanent magnetization when all external magnetic fields have been turned off. This feature adds complexity to the materials in the form of magnetic memory [15–18]. Interesting variations consider the interplay between magnetically soft and hard components [19, 20]. Since, still, basic relations are to be discovered and material descriptions must be worked out, we here mainly remain within the framework of magnetically soft materials.

Two major effects of magnetoresponsive behavior are frequently discussed in the literature. The first one is magnetostriction [21–28]. When the particles are magnetized by homogeneous external magnetic fields, they develop mutual magnetic interactions. Consequently, they press and tear on the surrounding elastic medium [11, 13, 29]. This leads to overall deformation. The second major effect is magnetorheological behavior [12, 28, 30–35]. That is, magnetization of the particles affects the overall static and dynamic mechanical moduli of the materials. Applications of these effects have been outlined and discussed, for example, in terms of magnetic valves and pumps [36, 37] or magnetically tunable damping devices [38–40]. Additional features comprise, for example, magnetically tunable conductivities, mainly electric conductivity [41–43] and heat conductivity [44].

The search for the underlying causes of these types of behavior brings us to some of the major challenges in the research on magnetic gels and elastomers. Optically, with increasing thickness, they are nontransparent. Therefore, revealing internal processes in bulk samples requires refined tools such as X-ray microcomputed tomography [45]. Next, the external magnetic stimulus acts on a microscopic scale, corresponding to the size of the particles. Yet, the response in terms of overall deformation or changes in rheological behavior are evaluated on a macroscopic scale. Thus, enhancing our understanding and control of these materials requires links across different length scales [46–48]. Fortunately, for many cases, the discrete nature of the polymer mesh does not need to be resolved. Instead, the elastic component can frequently be viewed as continuous. However, theories and simulations then must couple the discrete particle picture to a continuum description of the surrounding material for a microscopic, particle-based understanding of the underlying effects [49, 50].

**Fig. 1:**
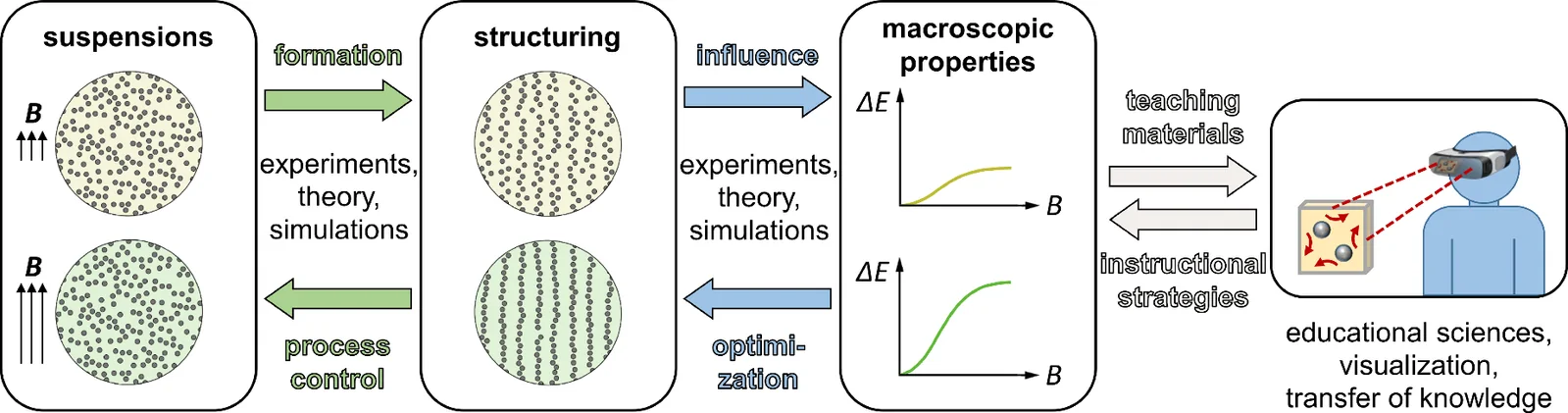
Illustration of the framework, goals, and workflow of combined research on structured magnetic elastomers and transfer of associated knowledge. Structures in magnetic elastomers are formed and imprinted, for instance, by applying strong external magnetic fields $\textbf{B}$ when they form from still fluid suspensions. Such and other microscopic processes are investigated in experiments, see Sect. 2, and in theory and simulations, see Sect. 3. Resulting microstructures affect macroscopic material properties, such as changes in mechanical moduli ΔE as a function of the applied magnetic field of magnitude *B*. Corresponding relations are revealed by experiments and associated data analysis, see Sect. 4, and by simulations, theoretical and further computational tools, see Sect. 5. Resulting knowledge, materials, and visualizations serve as a basis for educational sciences to investigate how knowledge about magnetic elastomers as well as basic principles of magnetism and elasticity are best transferred to an academic and a nonacademic audience, specifically school students, see Sect. 6. Goals are, for example, to develop instructional strategies of most beneficial presentation and transfer of knowledge, in addition to transmitting a general fascination for STEM subjects. Concerning materials science, goals are to identify ways of controlling the process of structure formation during material preparation, so that materials of optimized overall properties are generated

In the search for microscopic foundations of the overall behavior, it was noted that structural properties are important. Specifically, the positional arrangement of the particles plays a major role. Chain-like aggregates [5, 13, 30, 45, 51, 52] or more complex anisotropic elements such as rings or tubes [53, 54] can be locked into the materials by applying strong uniform external magnetic fields when generating the polymeric carrier medium. In a still fluid reactive suspension, before chemical crosslinking and formation of the permanent elastomeric matrix take place, the magnetized particles can rearrange into anisotropic aggregates, see the left two columns of Fig. 1. While maintaining this state, formation of the surrounding soft elastomer is initiated. Under such conditions, the anisotropic particle arrangements are permanently locked into the materials, because they are too large to move through the polymeric mesh. It was found that this process of structuring the magnetic elastomers during their formation can substantially affect their response to external magnetic fields. Examples concern magnitudes of magnetostriction [22, 26, 55, 56], the type of induced modes of magnetostrictive deformations [57–60], heat conductivity [61–63], or magnetorheological behavior [5, 14, 56, 64–67], see the center two columns of Fig. 1. When we wish to stress the internal presence of anisotropic particle aggregates or of more complex, organized particle arrangements, we refer to such magnetic elastomers as “structured magnetic elastomers”.

Still, magnetically induced internal restructuring is possible even in the finalized materials under application of external magnetic fields, against the elastic restoring forces of the carrier medium. Using optical observation [65] or X-ray tomography [14, 68, 69], rearrangements of the particles into chain-like, anisotropic aggregates from initially rather random distributions were revealed. Such structural rearrangements were correlated with changes in overall mechanical behavior [14].

All these observations make us conclude that the internal structure in terms of the positional arrangement of the magnetizable particles plays a dominant role in understanding and leveraging the material behavior. Identifying most beneficial structures and paths towards their realization is therefore a central part of current research, as indicated in Fig. 1.

On our way, we note that these systems involve a very illustrative and intuitive physical background. They combine two basic physical effects, namely magnetism and elasticity. Most of us have been familiar with these effects already from our childhood. Magnetized microscopic particles attract and repel each other like little magnetic toy beads or bricks that most children play with at some point. Likewise, basically every kid has experienced elasticity while playing with rubber bands or other soft materials. Magnetic elastomers combine both kinds of behavior.

Consequently, this intuitive background sets our idea to use magnetic elastomers as a vehicle to convey the fascination of these materials and of research in STEM fields in general to the nonacademic world, see the right panel of Fig. 1. At the same time, we wish to explore in the framework of our subject how transmission of knowledge generally works. More specifically, the question is, how a given realistic background and context, here provided by the research on magnetic elastomers, affects the transmission of knowledge and processes of learning of generic physical effects.

This question of teaching and learning is a very timely one in educational sciences [70–75]. Methods to analyze and quantify the process of teaching and learning as a whole or in parts comprise tools that record snapshots of the current state during the learning process over longer periods of time. Common tools are specifically developed questionnaires and their statistical analyses [76–78]. Over shorter periods, resolving aspects of the actual dynamics of acquiring new information and knowledge, learning persons are observed in real time. One specific technique involves eye-tracking apparatuses [79–87]. The paths of the eyes when exposed to new information such as diagrams are recorded and analyzed. Corresponding statistics, for example, of periods of focus on certain pieces of information or jumps between them help to judge the quality of the learning material and suggest ways of improvement. The context of magnetic elastomers can provide corresponding input for education research, even on the level of high-school education. Conversely, STEM fields can benefit from output of education research to reorganize modes of presentation for better transmission of research results, as indicated on the right-hand side of Fig. 1.

Together, building the bridge from fundamental and materials science to educational science, the field of magnetic elastomers and its extension to research on teaching and learning put forward many immediate, appealing, and burning questions. Three of those are formulated in the following way: Which structures of particle arrangement in magnetic elastomers lead to most requested results, typically meaning maximized effects of material behavior, such as magnetostriction and magnetorheology? How can such structures be realized during fabrication and which processes take place during structure formation? How can this topical background serve to best transmit knowledge about fundamental physical interactions, such as magnetism and elasticity, to the public outside academia, specifically school students, and how does the context of timely research support transfer of knowledge in general?

Obviously, answering such questions requires the combination of a broader range of interdisciplinary background and methodology, see Fig. 1. In the following, we provide five different perspectives that, together, lead to a more complete picture. The first one is experimental and focuses on the physical processes of structure formation, see Sect. 2. It addresses reactive suspensions of magnetizable particles on their dynamic path to elastic composite systems, while external magnetic fields induce the formation of anisotropic particle agglomerates. The second point of view likewise reveals dynamic processes of structure formation in magnetizable composite systems on a particle scale. Yet, it is theoretical in nature and can focus more specifically on the different involved interactions, see Sect. 3. Third, a more holistic point of view of materials science and engineering is concerned with synthesizing and measuring the properties of entire materials, see Sect. 4. More to that, it relates these properties to internal structural features. Fourth, scale-bridging simulations can confirm and immediately elucidate such relations, complementing the picture and acquiring quantitative predictive power. This type of approach can develop new material models to enhance theoretical descriptions but also to outline new routes of design, see Sect. 5. Finally, these elements are absorbed by education research that analyzes ways of transmission of associated knowledge to others, see Sect. 6. Specifically, the context of current research on structured magnetic elastomers provides a framework to convey generic physical phenomena such as magnetism and elasticity. Education research may reveal on the example of magnetic elastomers how a specific subject of current research may support the process of learning in general as well as transmission of knowledge in particular. Improving the presentation of research processes and results for selected target groups is one goal.

Each of the following sections is devoted to one of these five perspectives. In each case, we overview the state of the specific subfield, outline current challenges and potential ways of their solution, and append an associated outlook. Long-term goals are to reveal processes to prepare structured magnetic elastomers in ways that optimize their material behavior as requested and to identify modes of best conveying knowledge about them, as indicated in Fig. 1. Corresponding brief overall conclusions and a short perspective are provided in Sect. 7.

### Acknowledgments

We acknowledge support by the German Research Foundation DFG (Deutsche Forschungsgemeinschaft) through Research Unit FOR 5599 on structured magnetic elastomers, project nos. 511114185 and 535421963.

## Internal dynamics of magnetic particles during fabrication and magnetomechanical loading of structured magnetic elastomers—experimental studies

Konstantin Gerstenberger, Reza Azizmalayeri, Stefan Michel, Maximilian Lange, Nelly Pappermann, Günter K. Auernhammer, *Leibniz-Institut für Polymerforschung Dresden e. V., Dresden, Germany*

### Current state

As discussed above, earlier work concentrated on manufacturing and characterizing anisotropic magnetic elastomers as a whole. Applying strong uniform external magnetic fields during synthesis induces the formation of anisotropic particle arrangements. These structures become permanently embedded in the material as the solid, elastic polymer body forms [51, 88]. In recent years, there has been a particular focus on investigating how parts of these internal structures react to time-varying external magnetic fields [11, 13, 89–93]. This research is based on existing methodological developments [94–96] and their further refinement [97–99].

**Fig. 2:**
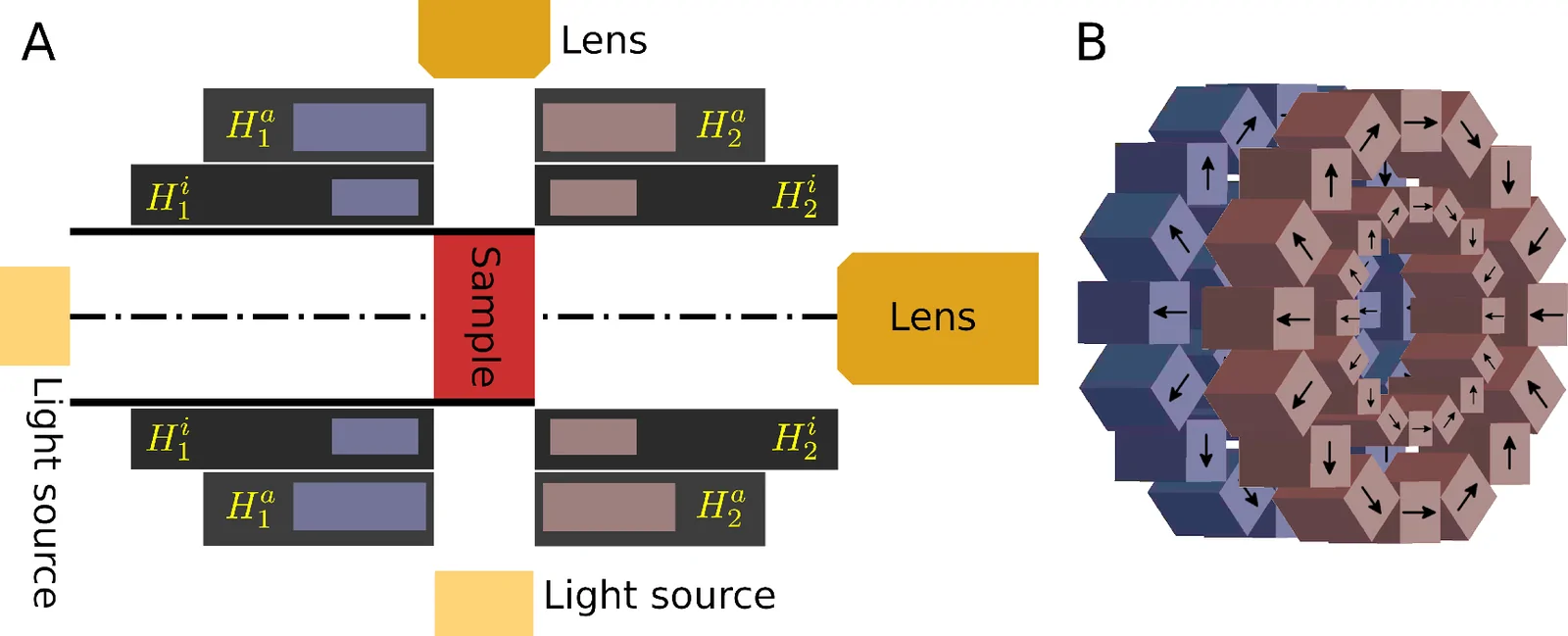
Sketch of an enhanced two-layer Halbach magnet array. Housing and rotary drive for the four rings of permanent magnets and the sample are not depicted. **a** Vertical cross section through the structure. The dash-dotted line shows the axis of rotation of the rings of permanent magnets and the sample. The four rings are labeled such that the upper index denotes the inner ($^i$) or outer ($^a$) ring, and the lower index denotes the left ($_1$) or right ($_2$) ring. **b** Oblique view. Two concentric rings in the same plane (same color) are referred to as a double ring. Specific arrangements of the individual magnets are not displayed to full detail for better visualization. The figure in (**b**) is adapted from Ref. [13], published under Creative Commons CC BY license

At low concentrations of magnetic particles, crosslinking in an external magnetic field results in the formation of linear particle chains. If an external magnetic field is applied to the finished crosslinked sample oblique to the chains, a magnetic torque acts on the chain structures [100, 101]. Using nonmagnetic marker particles dispersed in the elastomer to observe the mechanical displacement field showed that, in the final elastomeric state, the magnetic torque is balanced by an elastic restoring torque due to deformation of the surrounding elastic matrix [13]. Smaller chains can rotate, whereas longer chains begin to form wave structures and buckle. In particular, the rotation of smaller chains can be used to determine the local elastic modulus [13, 89, 92, 100, 101]. For example, this possibility was applied to determine frequency-dependent moduli in two-dimensional gels [89].

Analytical modeling and computer simulations have frequently been employed to quantify the underlying mechanisms. The interaction between single magnetic particles in an elastic matrix can be quantitatively described on a single-particle basis [11, 50, 91]. To this end, it was demonstrated that the displacement of particles within the elastomer is affected by the presence and induced displacement of nearby other particles. For this purpose, experimental observations were combined with explicit analytical calculations based on linear elasticity theory as well as finite-element simulations. Together, they promoted our understanding of resulting elastomer-mediated interactions [11, 102, 103]. Theoretical descriptions were also used to confirm the mechanism behind the buckling of chain-like aggregates in soft surrounding elastic matrices [13].

In three-dimensional investigations, confocal microscopy of fluorescent markers dispersed in the elastomer can be used to measure mechanical deformations when the amplitude of the external magnetic field is ramped up. Along with tomographic images, this method can prove and characterize the rotation of particles in magnetic fields and the deformation of the surrounding elastomer [90].

Additionally, for investigations of surface deformations on any reflective surface, a reflection method has been developed based on light sheets [104]. This method is particularly useful to study surfaces of larger, flatter magnetic elastomers. Preliminary experiments on two-dimensional particle arrangements revealed that applied magnetic fields cause significant local deformation in the films. Due to the induced mutual magnetic attraction, individual particle groups come into contact with each other. At the same time, the formation of larger particle aggregates is prevented by the deformation energy required for this process in the surrounding elastic matrix. Furthermore, it has been demonstrated that even slight rotation of the external field by five degrees leads to significant changes in the particle configuration [93, 105].

Lastly, we mention the ability to control mechanical bistability in spatially inhomogeneous, anisotropic magnetic elastomers [106]. Exploiting these instabilities in two- and three-dimensional systems can lead to a more effective use of the magnetostrictive effect and progress toward particle configurations that exhibit especially strong magnetostriction.

### Challenges and solutions

To reveal how to exert targeted influence on the manufacturing process of structured magnetic elastomers, especially the resulting positional arrangement of the particles, extended experimental investigations are necessary. Understanding how certain particle configurations arise and which factors influence this process is important. By investigating the interaction between aggregation and crosslinking dynamics, microscopic insights into the manufacturing process of structured magnetic elastomers can be obtained.

For this purpose, a strong, adjustable, homogeneous external magnetic field is needed. This can be achieved by arranging permanent magnets in a ring-like configuration, often referred to as Halbach array. Realizations in two and three dimensions are possible [13, 107, 108].

The orientation of each magnet within a ring according to its position is an analytically solved problem for two-dimensional magnets, also known as line dipoles [108], as well as for point dipoles [109]. However, it has been proven that the optimal arrangement changes when considering magnets of finite size [110]. Therefore, the existing Halbach arrangement should be viewed as a starting point for optimizing the homogeneity of the magnetic field. Finite-element simulations (FEM) based on Maxwell’s equations are used to calculate the magnetic field [111]. Starting from the idealized analytical solution, the magnets are allowed to tilt and rotate, and the variance of the magnetic field inside the Halbach array is evaluated. Applying the Newton–Raphson algorithm helps to find the optimal tilt and rotation angles of each magnet to create the most homogeneous magnetic field in a comparatively large three-dimensional volume. Additionally, using concentric double rings of permanent magnets in the same plane allows these rings to be rotated relative to each other. In this way, the strength of the magnetic field inside the double ring can be adjusted. For visualization, a graphical illustration of the set-up for the Halbach double rings is shown in Fig. 2.

Changing strength and orientation of the magnetic field can influence particle arrangements during the manufacturing process. The set-up provides additional access to the sample for further mechanical testing of its properties during the crosslinking process. In combination, analyzing such influences in a combined way can be leveraged to improve our understanding of the manufacturing process of structured magnetic elastomers. The dynamics of the particles inside the external magnetic field can be observed using cameras positioned between the magnet rings.

However, this method presents new challenges. Previous optical examination methods are either slow and allow for three-dimensional imaging, for example, conventional confocal microscopy, which has frame rates of only a few Hz [13, 90, 92]. Or they are limited to quasi-two-dimensional observation, such as transmitted light microscopy [11, 89, 91]. Various methods have been developed to overcome these obstacles.

To elucidate the internal structures in magnetic elastomers, their formation, and their response to external magnetic fields, the methods of high-resolution optical microscopy in terms of time and/or space [11, 13, 51, 88, 90, 91, 93, 112] and X-ray tomography are particularly well developed. Astigmatic particle tracking [98, 113–116] and fast confocal microscopy [97] are well established. Astigmatic particle tracking can be used especially for lower height differences between the particles. This method distorts the image in a conventional microscope using a cylindrical lens in front of a CCD camera. The shape of the imaged marker particles depends on their position along the beam axis. Monodisperse spherical marker particles appear as circular discs when in focus and as ellipses when outside this plane. The position along the beam axis can be determined by the ratio of the semi-axes of the ellipses. This allows three-dimensional trajectories to be determined from two-dimensional images at a high frame rate (several hundred Hz), making the method ideal for studying dynamic processes [98, 99, 117]. Originally developed for fluorescent marker particles, it can also be used in bright-field microscopy. To investigate particles with larger distances in all three dimensions, multicamera approaches can be integrated. The different positions of the high-speed cameras can be used to determine the movement of particles in space with three-dimensional particle tracking velocimetry [118–120]. In addition, fast confocal microscopy can determine the trajectories of marker particles in high concentrations [97].

In the context of sample manufacturing, it is crucial to recognize the significant impact of sedimentation on colloidal systems [121]. Also when magnetic elastomers are produced, gradients in particle concentration can result [45, 68]. One option to reduce the influence of sedimentation is to choose a high viscosity for the starting material. Then, only negligible sedimentation occurs before the elastomer is crosslinked [45, 68, 122]. Another way to suppress sedimentation is to use “simulated” microgravity. This is achieved by subjecting the sample to slow rotation under standard laboratory conditions [123]. Thereby, effects of sedimentation are averaged out. When sedimentation occurs at a sufficiently slow rate, simulated microgravity is an economically viable alternative to real microgravity.

**Fig. 3:**
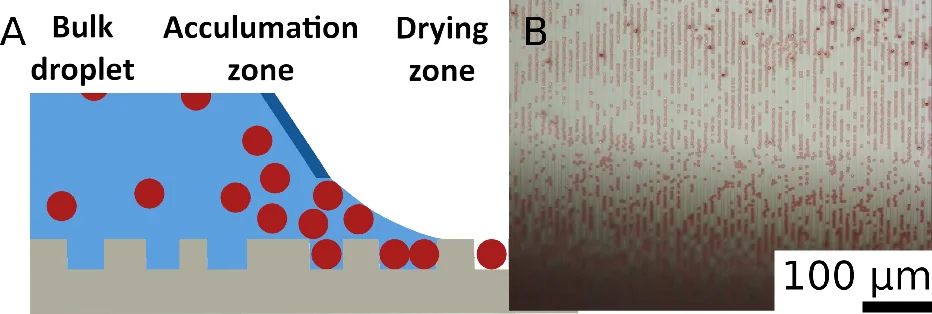
Method of capillary-assisted particle arrangement. (**a**) Sketch of the method. Through the combined effects of capillary forces and evaporation near the moving contact line, particles from the suspension are deposited into prefabricated structures of the substrate [124]. (**b**) Process of arrangement for approximately 5 μm thick magnetic particles using this method. The blurred area at the bottom corresponds to the accumulation zone in (**a**)

Lastly, realizing well-defined particle arrangements has been achieved through manual placement of particles [93]. However, this method has obvious limitations regarding the size of the arrangements and the particles. For two-dimensional arrangements, capillary-assisted particle deposition can be used to position significantly higher numbers of particles in a simple, easily reproducible way [125–128]. During this process, a particle suspension slowly dewets from a structured substrate. Capillary forces and evaporation near the moving contact line cause the particles to be deposited into the prefabricated pits of the substrate, see Fig. 3. For three-dimensional particle arrangements, 3D printing is a promising option. One typical challenge is to print particle-loaded materials. It has been reported that nozzle diameters exceeding the particle diameter by a factor of ten are necessary, which limits the resolution of the printed structures. Furthermore, a suitable magnetic field must be applied near the print head to achieve structuring [129].

Combining these techniques will provide a better understanding of the manufacturing process of structured magnetic elastomers, the potential structures that can be formed, and the properties of the resulting materials. Theoretical approaches used together with these listed experimental methods can serve to identify highly active particle arrangements for maximized material response and optimized material behavior.

### Conclusions and outlook

Summarizing, the variety of methods currently in use and their future extensions enable detailed investigations of the manufacturing process of structured magnetic elastomers and of their behavior in the finalized material on the particle scale. In combination, upgraded Halbach arrays allow to progress from studies and crafting of two-dimensional systems to larger three-dimensional samples. Additionally, corresponding set-ups serve to analyze particle migration and magnetomechanical response on the particle scale, see also Sect. 3, in spatially and temporally varying magnetic fields.

In combination, these approaches will allow to gain an in-depth understanding of the material class of structured magnetic elastomers and its manufacturing process. Specifically, they will enable the development of methods to control the manufacturing process and achieve requested structural and magnetomechanical properties. Together with scale-bridging studies, see Sects. 4 and 5, quantitative investigations of the role of selected particle structures for macroscopic material behavior represent a key focus.

Future results will answer questions about the physics of particle arrangements in complex rheological media, as well as the parameter adjustments required for manufacturing optimized magnetic elastomers. At the same time, new questions will arise when identifying and producing highly active two- and three-dimensional particle arrangements. Such configurations show large magnitudes of magnetomechanical response to moderate changes in external magnetic fields. Another remaining question is how this material class reacts on the particle scale to mechanical and magnetic dynamic stimulation, especially when the stimulation frequency is on the same scale as that of internal dynamical processes, and how these microscopic processes are reflected by the overall macroscopic response.

### Acknowledgments

We acknowledge support by the German Research Foundation DFG (Deutsche Forschungsgemeinschaft) through Research Unit FOR 5599 on structured magnetic elastomers, project nos. 511114185 and 535769078. We further thank Henrik Schmidt for contributing to Fig. 3.

## Magnetically induced particle dynamics and particulate structure formation in viscous and viscoelastic media—theoretical-numerical investigations

Henning Reinken, Markus Heiber, Lukas Fischer, Konstantin Zisiadis, Muhammed Muhsin Abdul Azeez, Andreas M. Menzel, *Otto-von-Guericke-Universität Magdeburg, Magdeburg, Germany*

### Current state

In theoretical investigations, the formation of structures and aggregates from magnetic particles has a long history. For simplicity and computational efficiency, a lot of focus has been placed on dipolar particles [130–135]. Effectively, a magnetic dipole is placed at the center of each particle, implying magnetic interactions between them. In attractive configurations, a repulsive interaction potential, such as Weeks-Chandler-Andersen [136], avoids unphysical overlap or collapse of the particles.

Resulting structures have been investigated theoretically and characterized at length. They comprise topologies of strings, rings, or branches [130–132]. Interesting variations arise from nonsymmetrically placed dipoles [137–139]. In reality, they could be realized by magnetic Janus particles that feature a magnetic cap on one part of their surface. Other variations address the shape, for example, cubical instead of spherical [140]. Also self-propulsion by activation through chemical fueling processes was considered [141–143]. Especially in three dimensions, more complex equilibrium structures such as tube-like arrangements arise [53, 144]. Generally, a big challenge in this context is the search for the actual energetic minimum in terms of the resulting configuration. Corresponding numerical search algorithms require large computational effort in view of the large set of positional and orientational continuous degrees of freedom [144]. The long-ranged, unscreened magnetic interactions pairwise couple all particles of the set to each other, which adds to the resulting computational effort. In reality, the angular range of attractive magnetic interactions increases when the dipolar approximation is dropped and the magnetization field within the particles is resolved [29] or higher-order magnetic moments are considered.

As mentioned in Sect. 1, we here focus on magnetically soft materials. That is, significant magnetization of the particles is only induced and maintained by an external magnetic field. When we start from a set of magnetizable particles and turn on an external magnetic field, the resulting magnetic interactions will generally induce spatial reorganization of the particles and structure formation. If the particles are surrounded by a continuous medium, such as a liquid or an elastic solid, this external medium will be set into motion by the moving particles. Resulting flows and displacements are long-ranged and couple all particles of the configuration [11, 103]. In surrounding elastic solids, elastic counterstresses arise upon particle displacements [91]. These counterstresses limit the potential range of reconfiguration.

Most theoretical works do not explicitly resolve interactions by flows [145]. Exceptions include studies based on smoothed particle hydrodynamics [146, 147], immersed boundary techniques [148], and lattice Boltzmann methods [149–151]. One challenge lies in ensuring the correct boundary conditions between the particles and the fluid.

Alternatively, approximative approaches are available. Specifically, rigid spherical particles of no-slip boundary conditions were considered, meaning that there is no relative velocity between the particles and their environment at their surfaces. In this case, couplings through external continuous media were described by analytical theory. On the one hand, these analytical approaches address interactions of particles of sufficient spatial separation. Then, expansions in the inverse distance between the particles are reasonable, both for suspensions of particles in fluids [152–154] and for particles enclosed by soft elastic solids [11, 49, 155, 156]. Fluid suspensions are usually treated as incompressible and at low Reynolds numbers, while solids are considered in the linearly elastic regime. On the other hand, analytical theories concern particles that are close to each other, in terms of lubrication interactions [152, 154].

### Challenges and solutions

Having means of theoretical description available for both significantly separated and adjacent particles, the challenge arises in bridging between the two scales [157]. We consider the scenario of magnetically driven particle aggregation. There, the individual particles may start from substantial separation distances. Upon attractive magnetization, they approach each other basically into contact. Indeed, during this scenario all ranges of separation distances must be covered. At the same time, the discrete particles must be correctly coupled to the induced flows and displacements in the surrounding continuous medium – a challenging task by itself. These flows and displacements are long-ranged and affect the structural formation process of all particles.

One solution to meet these challenges is given by phase-field-type descriptions. Instead of considering particles and fluids separately, the presence of the particles is indicated by a continuous scalar phase field. For instance, this field is “zero” outside the particles and “one” inside, while it smoothly interpolates between the two values at the boundaries of the particles. Specifically, we refer to the method of fluid-particle dynamics [158–161]. It is computationally efficient, illustrative, and in comparison with successful and effective particle-based methods [162] directly provides and works with the resulting flow and displacement fields. Since visualization is a major component of our considerations in view of interactions with education scientists, see Sect. 6, we introduce this method to the field of magnetic fluids and elastomers [163].

**Fig. 4:**
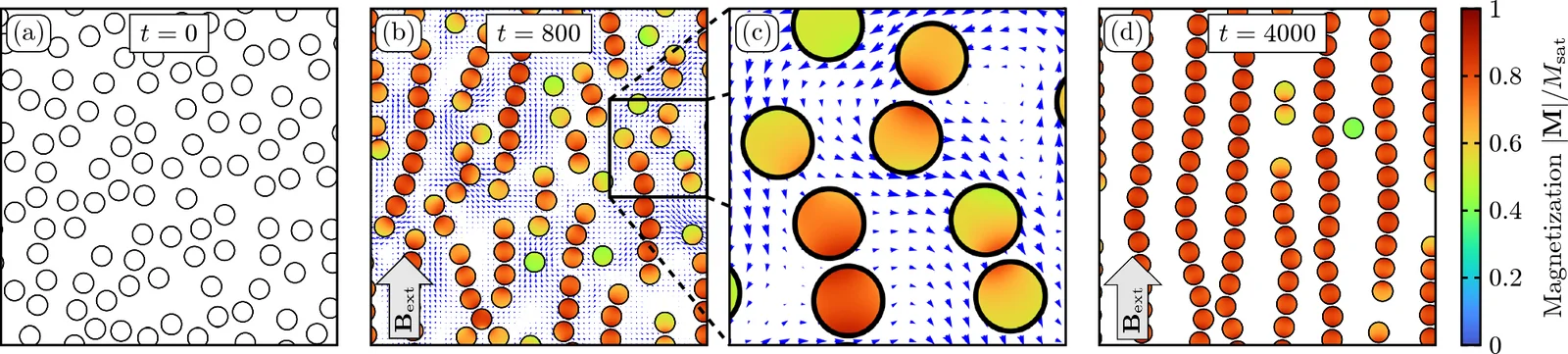
Structure formation resulting from an applied external magnetic field ${\textbf{B}}_{\textrm{ext}}$ [163]. (**a**) Magnetizable particles are initially randomly distributed in space at time t=0. (**b**) Snapshot at an intermediate time *t* after turning on the external magnetic field. Magnetization of the particles is indicated by the color scheme. Resulting magnetic forces between the particles lead to fluid flows as indicated by the blue arrows. (**c**) Close-up of part of the snapshot in (**b**). Mutual local magnetization of neighboring particles becomes significant. (**d**) After a longer time *t*, the particles have arranged into chain-like configurations

In essence, the particles are considered as parts of the fluid. Yet, the viscosity within the volumes of the particles is set to substantially increased magnitudes, so that these regions of the medium appear rather rigid [158]. Thus, the function determining the magnitude of the viscosity plays a role similar to a phase field. It varies continuously through the boundaries of the particles and thus equips these boundaries with a finite thickness. As major benefits, the coupling between the particles and the surrounding continuous medium is intrinsic to the method, and approach into virtual contact is numerically well supported. To stabilize the particles in their final configuration, effective repulsive interactions between them to represent volume exclusion must be introduced. A common solution is given by the Weeks-Chandler-Andersen potential [136].

Besides, when the particles approach each other, the approximation of mutual magnetic dipolar interactions between them breaks down. It is then appropriate to spatially resolve the magnetization and magnetic fields, inside and outside the particles. They result from an externally applied magnetic field [22, 91, 163, 164].

An example of the method in action is provided by Fig. 4. Here, we start from an initially random distribution of particles, see Fig. 4a. They are magnetized when applying an external magnetic field, inducing magnetic forces between them. As the snapshots of the intermediate states in Fig. 4b and c show, the resulting motion and flows reorganize the particles into chain-like configurations. Finally, Fig. 4d displays the completed chain formation after a longer time. The degree of organization in linear chain-like structures can be quantified by a chain-likeness parameter [163]. It measures how many particles have two near neighbors in comparison to those with more than two near neighbors.

We have recently extended the description to include elasticity. In this way, the dynamics of turning on and off an external magnetic field acting on the final elastic composite system [11, 13, 91] is described on the particle scale. It was demonstrated in experiments by optical and X-ray investigations that internal restructuring can occur. Initially well separated particles may jump into virtual contact upon magnetization due to the induced mutual magnetic attraction [68, 91]. Our final goal comprises nonlinear effects of elasticity to cover deformations of elevated magnitude.

Another challenge arises from the question, which configurations of particles one would actually prefer to obtain from the process of structure formation. In other words, once an elastic carrier medium has formed, which particle arrangements inside this medium are actually most beneficial from a practical perspective. In the context of magnetic elastomers, this aspect specifically concerns enhanced magnetostrictive and magnetorheological effects [5, 14, 22, 26, 55, 56, 64–67], see the overview in Sect. 1. Our first attempts therefore seek to maximize contraction or elongation along an external magnetic field in terms of magnetostriction. Analogously, the magnitudes of the magnetorheological effect shall be enhanced, that is, mechanical moduli under deformation shall be maximally increased or decreased when external magnetic fields are applied.

For a detailed answer to this question, a refined and quantitative approach of material modeling is necessary, see Sect. 5 below. One challenge lies with the mandatory scale bridging from the microscopic particle level up to the macroscopic behavior of the whole system. An initial solution in terms of a proof of concept can focus on reduced descriptions and appropriately chosen geometries.

In initial investigations, we focused on particles carrying induced magnetic dipoles embedded in a linearly elastic carrier medium. The particles are well separated from each other and from the boundaries. Consequently, we may, to lowest order, approximate the position of action of the force that each magnetized particle exerts on the elastic material as point-like. Elastic deformations result in the entire system by every such applied force.

**Fig. 5:**
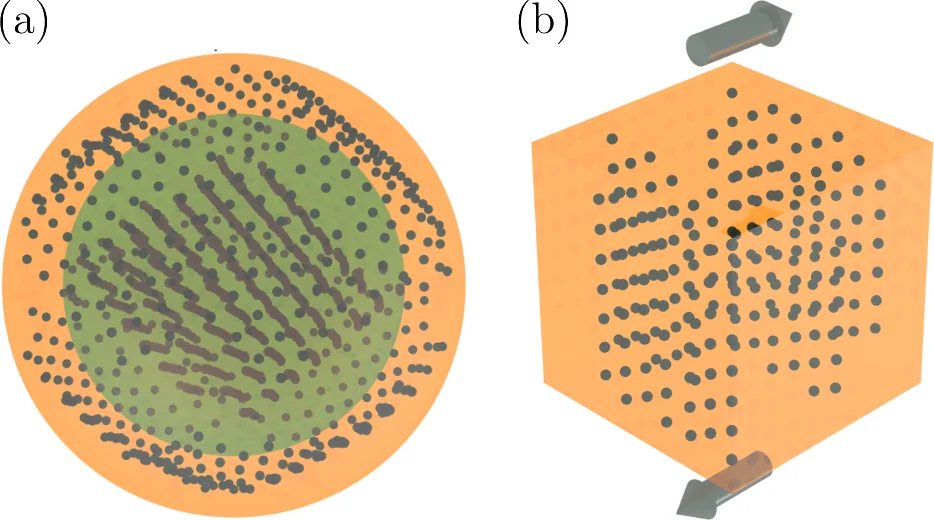
Through an adapted version of simulated annealing [167], configurations of magnetizable inclusions in a homogeneous, incompressible, linearly elastic medium were determined that lead to maximum response upon magnetization. An external magnetic field is considered to be applied in vertical direction and to magnetize the inclusions along this direction to saturation. (**a**) Magnetostrictive effect: The depicted spatial arrangement of the inclusions induces maximum vertical elongation of the sphere upon magnetization. In contrast to the more regularly structured inner volume (marked in green), the influence of the boundaries becomes evident near the surface. (**b**) Magnetorheological effect: A sheet-like arrangement of the inclusions leads to maximum increase of overall shear modulus of the composite system upon magnetization. Reproduced from Fig. 3A and Fig. 6C, respectively, of Ref. [167], published under Creative Commons CC BY license

Basic example systems to study magnetostriction are given by free-standing, soft, linearly elastic, nonmagnetic spheres containing the magnetized particles. The corresponding elastic Green’s function [26, 165], the fundamental solution of the underlying elasticity equation, quantifies the displacements at any position from the locations of the point-like particles and their exerted forces. Specifically, expressions for the amplitudes of overall uniaxial elongation or contraction can be derived from there [166]. The particle arrangements can be optimized for maximal elongation or contraction of the sphere along an external magnetic field. Orientations of the magnetic dipoles in this proof of concept were assumed as fixed by a strong, saturating, external magnetic field. The large number of degrees of freedom associated with the particle positions is highly challenging [130, 144]. Relying on an adjusted version of the statistical computational optimization algorithm of simulated annealing provides a proven solution [167]. Effects of the boundaries of the sphere were clearly visible, see Fig. 5a.

The algorithm was further transferred to identify optimized particle arrangements for enhanced magnetorheological effects [167]. Cubical incompressible systems were considered under affine uniaxial stretching and shear deformation. Structures for both maximal increase or decrease of static mechanical moduli were identified [167]. Mostly, sheet-like arrangements of internal hexagonal order were found to lead to maximum changes in moduli, see Fig. 5b. The orientation of these sheets depends on the considered geometry.

In a second step, the magnetically induced overall change in static mechanical moduli was further enhanced. To this end, the particle arrangement was simultaneously optimized for two effects. Magnetizing the particles along one direction, the overall modulus shall maximally increase. Magnetizing the particles along another, perpendicular direction, the overall elastic modulus shall maximally decrease. In combination, the relative change in modulus is significantly enlarged when switching between the two perpendicular field orientations [168], instead of just switching from a nonmagnetized to a magnetized state.

### Conclusions and outlook

In summary, theoretical methods are developed to quantitatively describe restructuring and the formation of particle aggregates under magnetization in continuous environments. Specific tasks involve the coupling between discrete particles and the continuous media. Flows and displacements are induced that lead to additional, long-ranged interactions between the particles and affect the resulting structures. The particles can approach each other from significant separation distances basically into contact, which introduces further challenges. A way to handle these challenges is given by phase-field-type simulation approaches. For this purpose, the fluid-particle method [158] is introduced to the field of magnetic fluids and elastomers by extending it accordingly.

In the future, this basis allows us to analyze effects that contribute to magnetically induced structural aggregation processes. They become relevant when generating structured magnetic elastomers. Dynamics in the final elastic product can be described as well.

At the same time, desired structures in terms of particle configurations were identified by initial scale-bridging theoretical and simulation approaches. Their goal is to maximize magnetostrictive and magnetorheological effects. A first proof of concept has been provided. Such strategies can be combined with more refined and quantitative ways of material modeling, see Sect. 5.

The question is how to drive the formation process on the particle scale in a way to end up with the requested configurations that lead to the desired macroscopic behavior. Answering and promoting this question will be one of our major topics in future research, together with our collaborators. One key aspect may consist of varying the magnetic field intensity and orientation during the structural formation process [93, 169]. In the longer term, improving and adjusting methods of 3D printing could enhance realization of tailored structures, at least on slightly larger length scales [170, 171].

### Acknowledgments

We acknowledge support by the German Research Foundation DFG (Deutsche Forschungsgemeinschaft) through Research Unit FOR 5599 on structured magnetic elastomers, project nos. 511114185 and 535543971.

## Process-controlled fabrication of structured magnetic elastomers, X-ray tomographic structure analysis, and macroscopic magnetorheology

Nils Magin, Stefan Odenbach, *Technische Universität Dresden, Dresden, Germany*

**Fig. 6:**
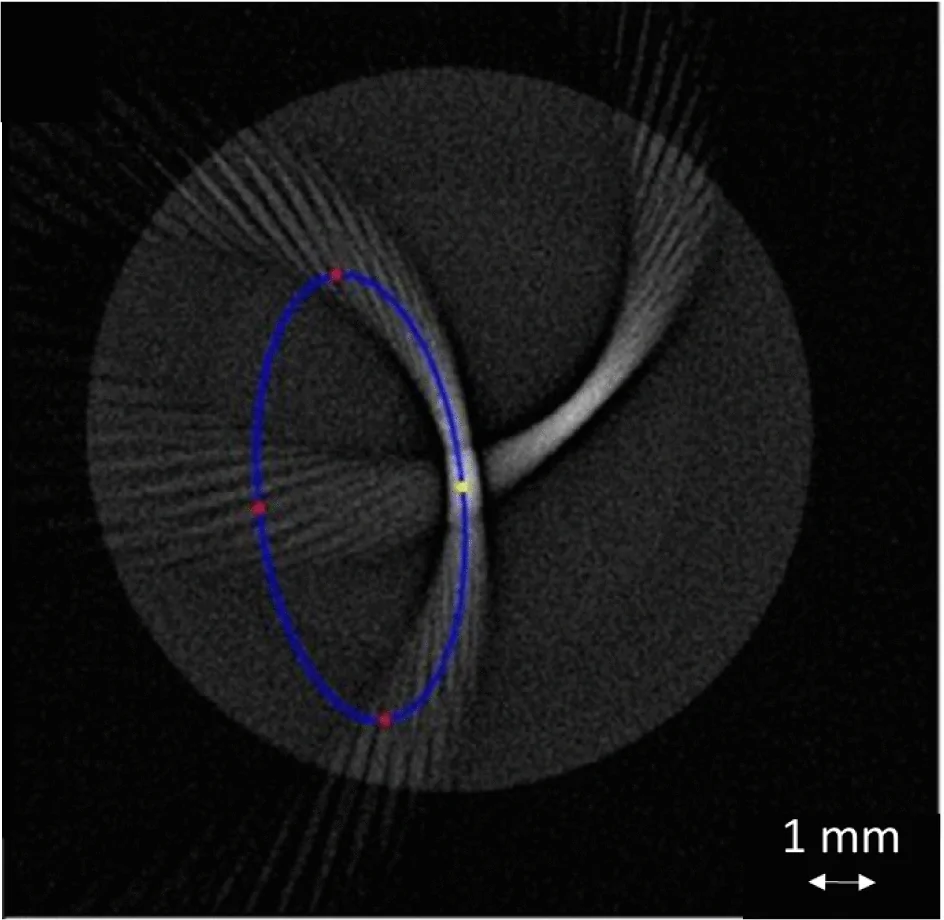
Results of image processing showing crossing, arc-shaped artifacts caused by the motion of two particles moving during the time of acquisition of a tomogram. Their motion here resulted from sedimentation. An ellipsoid was fitted to one arc of the artifacts. From here, the particle coordinates can be extracted and subsequently the trajectories can be determined. Reproduced from Fig. 1f of Ref. [185], published under Creative Commons CC BY license

### Current state

Over the past few decades, X-ray tomography has become a standard method for structural characterization in materials science [172], including the field of magnetic elastomers [14, 45, 53, 54, 68, 90, 92, 173–178]. Alternative methods for structural analysis of magnetic elastomers cover classical microscopy [179–181], which initially focused on two-dimensional image acquisition. Besides, small-angle neutron scattering (SANS) [182, 183] concentrated on statistical data recording. X-ray tomography is a nondestructive process that enables acquisition and reconstruction of three-dimensional information on the sample.

The method is highly suited to investigate the spatial arrangements and structures formed by magnetic particles in magnetic elastomers. External magnetic fields can induce the formation of such particle structures in reactive suspensions before crosslinking and the formation of a permanent elastomer take place. Once the surrounding soft elastic solid is established, the particle structures are locked in [5, 7, 13, 30, 53, 92, 177, 184].

Resulting structures can be broadly categorized into three distinct groups based on three-dimensional X-ray tomograms. Particles aligned in a linear fashion along the magnetic field during crosslinking are referred to as “chains” [45]. Structures consisting of multiple “chains” lying parallel to each another and forming bundles are here referred to as “rods”. In the case of more complex structures, when it is difficult to distinguish between individual chains, the term “cluster” is applied [53].

The type of resulting structure depends on various parameters. First, this is the magnetic field applied during structure formation, followed by the particle concentration and the particle size distribution. When using identical magnetic fields but increasing the particle concentration, thin “chains”, then “rods”, and subsequently more complex “canyon-like clusters” are observed [45, 53]. Moreover, at a constant particle concentration, an increase in magnetic field strength leads to an increase in the cross-sectional area of the structures, accompanied by a decrease in the number of “chains” or “rods”. The minimum required magnetic field to form such particulate structures is found to decrease with decreasing particle size [45].

Additionally, high-resolution tomograms and techniques of digital image processing can be used for configurational analyses on the individual-particle level. They provide further information, including the number of particles in chains [177] or the three-dimensional pair correlation function (PCF) [174]. The PCF quantifies the probability of finding another particle at a given distance in a given direction when measured from a first particle. It is used to identify anisotropies within the structures, and potentially to conclude on underlying interactions between particles [174].

Identifying and labeling individual particles by their shape, they can be tracked across multiple tomograms under modified external conditions. Examples are alignment of nonspherical particles with external magnetic fields [90], or displacements under external mechanical forcing [14]. In this way, alterations in the configurations of the individual particles correlate with macroscopic changes in behavior of the whole sample. For this purpose, magnetically induced changes in macroscopic mechanical properties, that is, the magnetorheological effect, can be measured, for example, using tensile testing devices [15, 92]. Accordingly, correlations between microstructural parameters and the macroscopic magnetorheological behavior were identified [174].

### Challenges and solutions

It is challenging to reveal explicit links between microstructural properties and the macroscopic material behavior. One way to advance our understanding is to focus on different stages of structure formation and their relation to the overall properties. To this end, the spatial arrangement of the particles must be recorded at each stage together with the material response.

The most significant challenge in this context is that typical processes of particulate structure formation in a not yet fully crosslinked elastomer last only minutes or seconds, depending on the average particle size and viscosity of the material [93, 186]. Conversely, acquiring a high-resolution tomogram to explore the state of the structure takes approximately an hour [187]. Consequently, structure formation and the measurement process occur on completely different time scales.

**Fig. 7:**
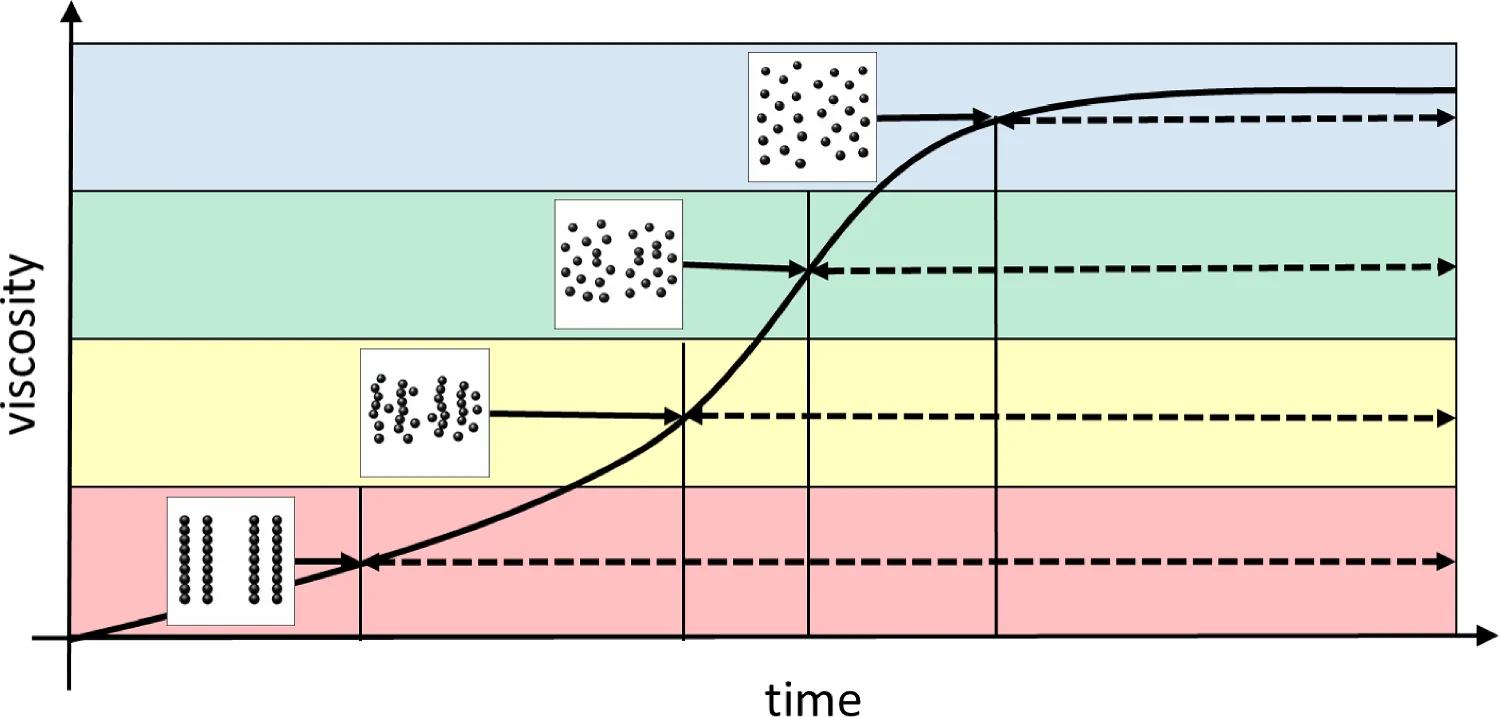
Schematic illustration of the methodology to acquire X-ray tomograms of intermediate stages of structure formation in magnetic elastomers by preparing and analyzing “irreversibly delayed states”. While crosslinking in a sample proceeds, the effective viscosity increases (solid line). The process is divided for illustration into four regions as marked by different colors. First, in the low-viscosity region, the sample is still uncured and enclosed particles have high mobility (red). Next, viscosity increases and particle mobility decreases (yellow and green). Finally, the sample is basically cured (blue), so that net particle motion becomes negligible. Starting from different times during the crosslinking process for different samples, a strong homogeneous external magnetic field is applied. The lengths of the dashed lines represent the durations of magnetic field application. Associated boxes qualitatively illustrate expected resulting structures in the final samples at low particle concentration. Depending on the starting times of applying the magnetic fields, the structures show pronounced chain-like configurations, shorter chains, or only rather isotropic configurations. Overall, from analyzing the particle configurations in the resulting samples containing the different “irreversibly delayed states”, the process of structure formation can be reconstructed

Therefore, it is not straightforward to observe the process of structure formation directly inside the not yet fully crosslinked samples while the structures are forming in the presence of an external magnetic field. Moreover, moving particles during tomographic recording, resulting from the much longer times in data acquisition when compared to particle dynamics, lead to arc-shaped artifacts in the final tomograms [185, 187]. Yet, what at first glance appears disturbing, can be deliberately exploited to analyze the underlying dynamics of structure formation, see Fig. 6. Resulting “blurring” [188] in the tomograms is specifically used to conclude on the motion of the particles while recording the data. In the context of multi-particle systems, it is imperative to start developing corresponding tools of analysis using particle concentrations as low as possible. This approach enables the accurate assignment of tracks of blurring to specific particles and minimizes crossings of such tracks.

To solve the problem of mismatched time scales and to nevertheless be able to relate the different stages of structure formation to overall material behavior, one strategy focuses on the concept of intermediate states of structure formation locked into the elastic materials. We distinguish between two opposing concepts. One leads to “permanently unfinished states”. It means that when an external magnetic field is applied to structure the particle arrangement while preparing the materials, crosslinking of the surrounding elastomeric confinement is initiated and finished before the structuring process has come to an end. In this way, the current particle arrangement is permanently locked into the material. Practically, this is achieved by quickly performing the crosslinking procedure of the elastomer while the structure is still at an intermediate stage [189]. Producing such samples requires precise control of the fabrication process. This objective can be accomplished through a variety of methodologies. An illustrative example encompasses a sudden increase in irradiation by UV light to complete sudden crosslinking [189]. However, this method presents additional technical challenges regarding the selection of a polymer system.

In order to circumvent these technical challenges, an opposing strategy is to exploit the gradual increase in viscosity of the polymeric carrier medium during the regular crosslinking process. Instead of applying the magnetic field from the start, it is only turned on to induce structure formation at later stages of crosslinking. Due to the then already elevated viscosity and thus reduced particle mobility, the process of entire structure formation cannot be finished in time, before the crosslinking density has become too large. Such procedures therefore likewise lock in intermediate stages of structure formation. They correspond to “irreversibly delayed states”, because application of the external magnetic field simply starts too late so that the structuring process cannot be completed in time.

Generating a row of samples and applying the magnetic field to induce structure formation at successively later times allows to conclude on the process of structure formation in magnetic elastomers in a rear perspective, that is, from the final to earlier stages. Each resulting sample then contains a certain intermediate state of structure formation. Since these structures are permanently locked into the different samples, they can well be explored using X-ray analysis without any issues of time constraint. Moreover, the overall mechanical properties can be measured and related to the different stages of structure formation to provide a complete picture. A schematic illustration of this approach is shown in Fig. 7.

Besides this major conceptual challenge and its solution, there are a few additional technical ones. First, sedimentation during sample preparation and structure formation must be addressed. From tomographic images, the influence of gravity is directly assessed [45, 177, 189]. In evaluating the resulting particle configurations, areas of obvious effects from sedimentation are identified. Solutions to reduce the impact of sedimentation include elevated crosslinking times [45, 174] or emulated microgravity by sample rotation [123].

Additional requirements must be met by the particles. They must be of sizes that are sufficient for the resolution of the tomographic set-up. Sieving serves to address this point [173]. Moreover, irregularly shaped particles are more reliably matched to themselves when tomograms of the same sample but at different magnetization are compared to each other [14]. Yet, at the same time, irregularly shaped particles present significantly larger challenges for simulations to develop a more complete understanding of the underlying processes, see Sects. 3 and 5. Using slightly ellipsoidal particles may provide a reasonable compromise in the future.

A specific further challenge related to the apparatus itself concerns the application of magnetic fields during data recording. The radiation source is exposed to the magnetic field of the electromagnet. Outside its poles, the electromagnet has a highly inhomogeneous magnetic field. The fundamental principle behind an X-ray source is the generation of X-rays through sudden deceleration of electrons. Generally, the motion of electrons is influenced by magnetic fields, which would modify the radiation characteristics of the set-up [190]. To minimize associated effects, it is necessary to shield the source.

### Conclusions and outlook

In summary, X-ray tomography is a reliable and fundamental experimental method to reveal particulate structures and processes of their formation in magnetic elastomers. A number of methodologies were proposed to overcome the time-scale discrepancy between data acquisition and the sequence of structural reorganization. These include evaluations of artifacts resulting from particle motion during data acquisition. Moreover, they comprise the fabrication and analysis of samples of fixed structures representing intermediate states. It is evident that both methods support a more profound understanding of the underlying processes of structure formation in magnetic elastomers.

As a future perspective, integrating magnetic exposure of samples within the tomographic apparatus in conjunction with a force-measuring apparatus will facilitate in-situ measurements of magnetorheological effects concomitant with the acquisition of tomograms. Such an integration will allow for a combined analysis of microscale particle behavior together with macroscopic material properties. More immediate correlations between structure formation, structural response, and resulting overall effects could be drawn.

Corresponding methodologies will support the targeted production of magnetic elastomers with specific requested properties. Systems of low particle concentrations are essential for fundamental research, while those with high loadings are important for practical applications. Augmenting the radiation output of the X-ray source will further ameliorate the potential of the experimental approach.

### Acknowledgments

We acknowledge support by the German Research Foundation DFG (Deutsche Forschungsgemeinschaft) through Research Unit FOR 5599 on structured magnetic elastomers, project nos. 511114185 and 535746395.

## Data-based scale-bridging simulation of structured magnetic elastomers

Heinrich T. Roth, Karl A. Kalina, Markus Kästner, *Technische Universität Dresden, Dresden, Germany*

### Current state

Material modeling is a cornerstone of solid mechanics. In recent years, research focus has increasingly shifted towards actively tunable materials [191], such as magnetic elastomers, which exhibit strong magnetomechanical coupling.

**Fig. 8:**
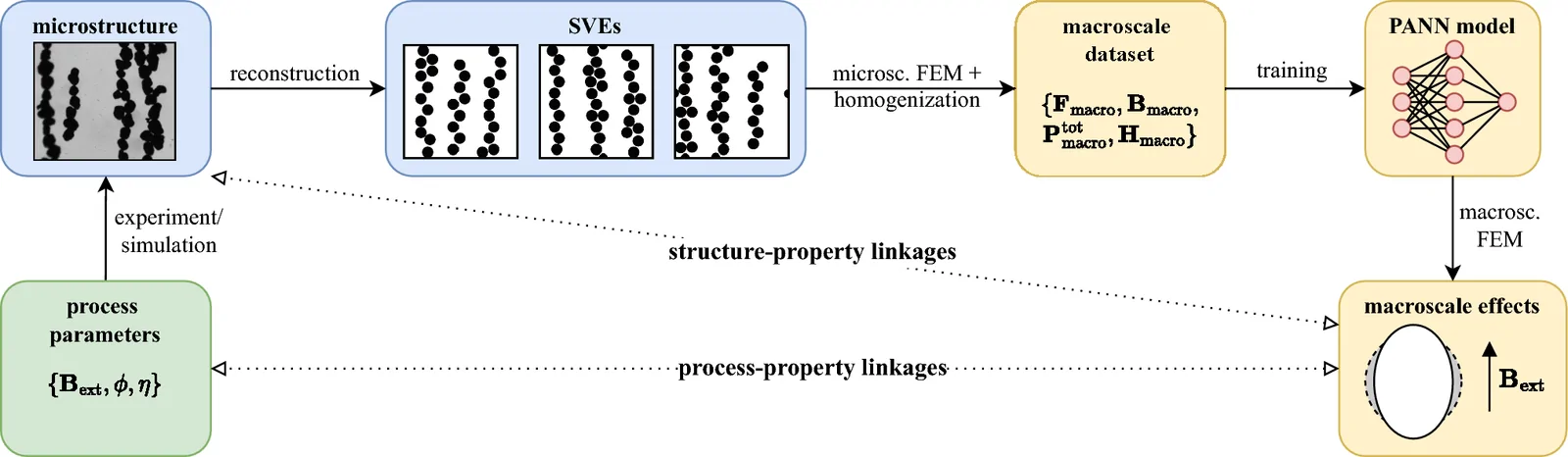
Framework for identifying structure–property and process–property linkages. Microstructures generated from a given set of process parameters are reconstructed into statistical volume elements (SVEs). From these, macroscopic data consisting of the deformation gradient, magnetic induction, total first Piola–Kirchhoff stress tensor, and magnetic field for the training of a macroscopic physics–augmented neural network (PANN) model are generated. The trained model is applied to simulate macroscopic effects

Simplified microscopic simulations allow insight into the mechanisms in magnetic elastomers. They show that three particles arranged in a chain-like particle arrangement move closer together under a magnetic field applied along the chain direction and move apart under a transverse field. If the central particle is sufficiently laterally offset, the opposite response occurs [192]. Furthermore, the magnetorheological effect of a cell with an ideal particle chain can be up to four times larger than that of a cell with randomly distributed particles [193]. Although these simulations provide qualitative insight and demonstrate the potential of structured magnetic elastomers, they are insufficient for quantitatively predicting macroscopic behavior. The latter depends on microscale interactions, specimen geometry, and boundary conditions. Numerous approaches were proposed to model the behavior of magnetic elastomers and are commonly classified as microscopic or macroscopic, depending on the resolved length scale [194, 195].

Microscopic models resolve individual particles and are therefore either computationally prohibitive or rely on restrictive assumptions on microstructure and material behavior. For instance, discrete particle models frequently assume low volume fractions of particles and dipolar interactions between them [196–198]. Continuum formulations resolving the particles allow for arbitrary particle geometries and volume fractions [164, 199, 200], but remain very expensive for simulations of realistic sample sizes. Homogenization approaches reduce the computational cost by solving the microscale problem on a representative volume element (RVE) [201–203], or by averaging over multiple statistical volume elements (SVEs). In coupled multiscale schemes [204, 205], however, the microscale boundary value problem must still be solved at each macroscopic integration point, leading to high computational costs.

Genuinely macroscopic continuum models treat the materials as homogeneous and do not resolve individual particles. Thus, they are computationally efficient. Corresponding phenomenological models [206–208] are calibrated using experimental data, but this can introduce dependencies on the sample geometry. Decoupled multiscale schemes [209, 210] overcome this issue by calibrating a macroscopic model based on homogenized microscale simulation data. In this way, they capture effective microscale behavior, while simultaneously they significantly reduce computational cost compared to coupled schemes.

### Challenges and solutions

Developing macroscopic continuum models trained on homogenization data is challenging. Existing models [211, 212] can describe isotropic magnetic elastomers but offer limited flexibility. Extensions to structured magnetic elastomers are nontrivial. For purely mechanical problems, neural network models with embedded physical constraints have been successfully applied in multiscale schemes [213]. Recently, a physics-augmented neural network (PANN) model was proposed in two dimensions for isotropic magnetic elastomers, which exhibit direction-independent properties [214], and then adapted to simply structured magnetic elastomers in three dimensions [215].

One strategy to relate process parameters of structure generation and microstructures themselves to macroscopic effects is outlined in Fig. 8. For a given set of system parameters, microstructures of magnetized particles emerge in experiments or simulations, see Sects. 2–4. Relevant parameters include, for example, the external magnetic field ${\textbf{B}}_{\textrm{ext}}$, particle volume fraction ϕ, or the viscosity of the noncrosslinked initial carrier fluid η. Resulting microstructures are reconstructed into multiple periodic SVEs that capture the effective response. A set of magnetomechanical loads is applied to each SVE. The microscopic boundary value problem is solved using the finite-element method (FEM). Results are homogenized, that is, averaged over the SVEs, to obtain a macroscopic dataset which is used to train a physics-augmented neural network (PANN). The trained PANN serves as the material model in macroscopic finite-element simulations. Accordingly, macroscopic effects, such as magnetostriction, and their relation to the underlying microstructure and process parameters during generation can be quantified.

Several challenges must be addressed to implement this procedure. First, particle positions and shapes must be segmented from microscopy images and X-ray tomographic data. This is particularly difficult in dense clusters, where many particles are very close or even in contact, making it difficult to distinguish single large particles from multiple smaller ones. A viable approach is to increase contrast followed by watershed segmentation [216]. Large, concave regions can then be split into convex components, as they likely represent multiple particles. After detection, particle shapes must be parameterized. The simplest assumption are spherical particles, characterized by center positions and radii. For particles with high aspect ratios, ellipsoidal representations may be required. The detected particles are then replaced by the corresponding idealized shapes while suppressing overlap. This can be achieved through numerical optimization that minimizes a loss function based on squared overlap distances. For ellipsoidal particles, efficiently evaluating interparticle distances poses an additional challenge.

**Fig. 9:**
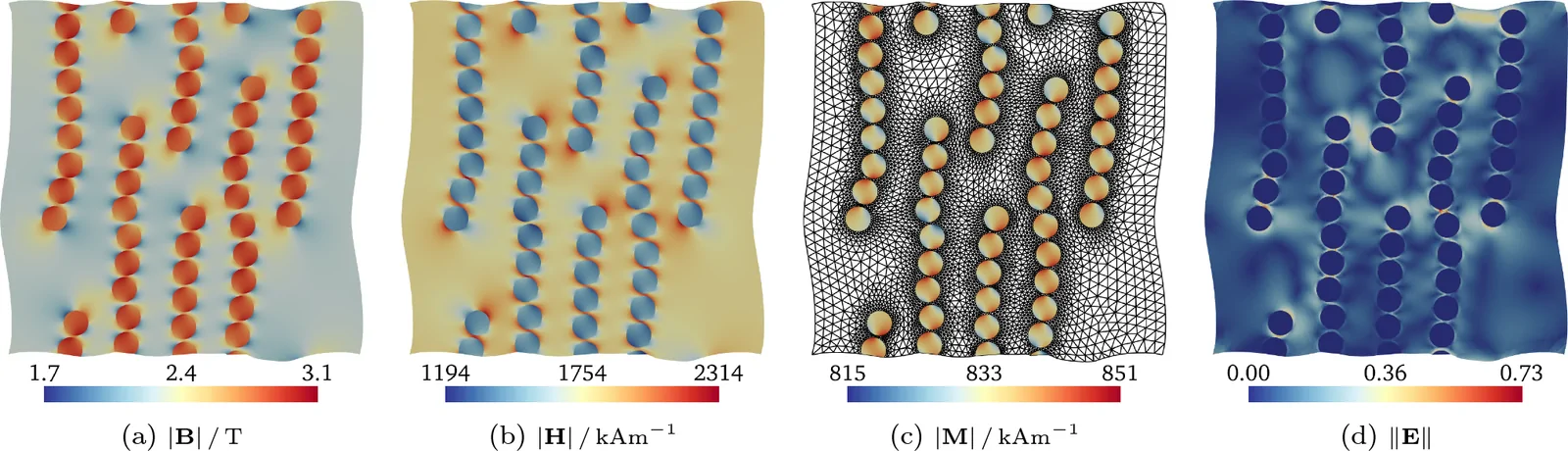
Deformation of a two-dimensional, initially square-shaped area element under a macroscopic magnetic induction of magnitude $2.37\,\textrm{T}$ and zero macroscopic deformation. We display the magnitudes of the microscopic field quantities (**a**) magnetic induction $\textbf{B}$, (**b**) magnetic field $\textbf{H}$, (**c**) magnetization $\textbf{M}$, and (**d**) Green–Lagrange strain tensor $\textbf{E}$. The magnetization is displayed only for the particles, as the magnetization vanishes in the surrounding nonmagnetic carrier medium

For a given parameterized microstructure, multiple smaller periodic SVEs with similar effective properties must be generated. These properties are typically assumed to depend on purely geometric microstructure descriptors [217, 218]. Reconstruction is formulated as an optimization problem that minimizes the deviation between descriptors of the reference and reconstructed microstructures. Additional constraints, such as minimum interparticle distances, can be enforced via penalty terms. For differentiable descriptors, gradient-based optimization is applicable. A common choice is the two-point correlation function [217], for which a differentiable formulation exists for ellipsoidal particles [219]. While effective for small two-dimensional microstructures in structured magnetic elastomers, reconstruction quality deteriorates for larger structures. Convergence slows down and computational cost increases, particularly for high area fractions and with enforced minimum distances. The quadratic scaling of pairwise distance calculations with the particle number can be mitigated by restricting computations to nearby particles using Verlet neighbor lists [220]. As an alternative descriptor, the average microscopic structure factor can be used. Together with the particle volume fraction and susceptibility, it determines the effective susceptibility tensor, which governs the direction-dependent initial slope of the magnetization curve [221]. Although based on dipole assumptions and limited to magnetic effects, it may still provide a useful estimator of macroscopic behavior. An alternative approach is the sequential addition and migration algorithm [222], which has shown good accuracy and efficiency for microstructures with long cylindrical inclusions.

Once the SVEs are defined, training data for the PANN is generated. The training data consist of effective deformation gradients and magnetic inductions prescribed to the SVEs, together with the corresponding effective stresses and magnetic fields obtained from microscopic FEM simulations and homogenization. First, microscale material models for the elastomer and the particles are specified [193]. Next, a set of macroscopic deformation gradients and magnetic inductions to be applied to the SVEs is selected. To obtain a more uniform coverage of the input space and to avoid clusters and large gaps, Latin hypercube sampling is employed instead of random sampling [214]. In addition, load states that would produce nearly identical inputs to the neural network are removed, as they do not provide additional information for training. The sampling boundaries must be chosen carefully. On the one hand, they should be sufficiently small to avoid load states for which the microscopic FEM simulations fail to converge. On the other hand, they must be sufficiently large to encompass all load states that may occur during the PANN application in macroscopic simulations, thereby avoiding extrapolation. Since the range of deformations and magnetic fields encountered during macroscopic simulations is generally not known a priori, the sampling boundaries may need to be refined iteratively [213].

Due to strong nonlinearities, the microscopic boundary value problems are solved iteratively with Newton–Raphson schemes. Linearized systems are solved at each step of iteration. For large systems, iterative solvers with suitable preconditioners are essential. Line search strategies and reduced load step sizes can be used to prevent nonphysical updates and improve robustness [223]. Many elastomers exhibit nearly incompressible behavior. In displacement-based FEM formulations, this can lead to an artificially stiff response, a phenomenon known as volumetric locking. This issue can be avoided by using mixed formulations, in which additional primary field variables associated with volumetric deformation are introduced [224, 225]. A regularization approach improves well-posedness in formulations using magnetic vector potentials [226]. Representative microscopic results are displayed in Fig. 9. The pronounced spatial variation of the magnetization within the particles confirms that dipole models become insufficient with decreasing particle separation. Spatial resolution of the magnetization fields becomes mandatory.

Once data has been generated, it is used to train a PANN that incorporates physical constraints. The conditions of objectivity and material symmetry are enforced by using specific invariants as input for the model [215, 227]. These invariants depend on the anisotropy class of the material, that is, the set of symmetry transformations under which the material response remains unchanged. For transversely isotropic structured magnetic elastomers, only one structure tensor that is invariant with respect to rotations around the chain direction is required. More complex anisotropies can be detected automatically during training [228]. An irreducible integrity basis can be identified for each class [229]. Several additional conditions can be met by correction terms in the PANN, for example, vanishing stress and magnetization in the unloaded state [214]. Further constraints on the magnetization are more difficult to enforce. For instance, weak enforcement of a bounded, monotonic, and concave magnetization curve can be achieved using extra loss terms [215]. Weighting these terms requires to balance accuracy in stress and magnetization against satisfying physical constraints. This weak enforcement does not guarantee that the conditions are satisfied. Strong, that is, a priori enforcement of the conditions on magnetic saturation remains an open challenge.

Once trained and validated, the PANN can be used in macroscopic FEM simulations to predict magnetostrictive and magnetorheological effects. Training parameterized PANNs [230, 231] on various sets of process parameters provides a direct mapping from parameters to effective material behavior. With the forward model at hand, inverse problems can then be addressed using gradient-based optimizers [232]. For example, one goal is to identify suitable process parameters to generate microstructures for desired or maximized magnetomechanical coupling effects of structured magnetic elastomers.

### Conclusions and outlook

Linking microscale structures to macroscale behavior in magnetic elastomers via an effective material model is a task of substantial current and future interest. Particle arrangements must be quantified and SVEs be generated, while limitations of current reconstruction algorithms must be addressed. Homogenization techniques applied to SVEs provide training data for PANNs, resolving numerical stability challenges in microscale FEM. PANNs are trained and then applied in macroscopic FEM simulations. Parametrized PANNs further enable mapping from process parameters of microstructure generation to macroscopic effects. On this basis, strategies of optimizing desired material responses by adjusting process parameters during microstructure generation can be developed by addressing the inverse problem.

Future work can comprise the automatic inversion of process–structure–property relations, which could be implemented via Bayesian optimization [233]. Incorporating dynamics [234] would require adapted homogenization schemes [235] and a magnetoviscoelastic model accounting for dissipation [208, 236]. Further extensions could include magnetically hard particles [237] and applications to 3D-printed magnetic elastomers [238].

### Acknowledgments

We acknowledge support by the German Research Foundation DFG (Deutsche Forschungsgemeinschaft) through Research Unit FOR 5599 on structured magnetic elastomers, project nos. 511114185 and 535639916.

## Modeling the effect of authenticity and perceived complexity on situational interest, interest in scientific activities, and cognitive learning outcomes in the context of magnetic elastomers

Deniz C. Senel, Bianca Watzka, *RWTH Aachen University, Aachen, Germany*

### Current state

Designing authentic learning environments has become increasingly important in science education, as they can foster students’ interest, engagement, and understanding of science [239, 240]. Beyond learning scientific facts and concepts, students should develop an understanding of how scientific knowledge is generated. In this sense, authenticity refers to the extent to which learning activities reflect essential elements of real scientific practice – such as formulating research questions, collecting and interpreting data, and drawing evidence-based conclusions [72, 241]. Learning environments that incorporate such authentic features may therefore support both conceptual understanding and students’ understanding of how science works. Early work emphasized authenticity as a general design principle intended to bridge the gap between school science and professional scientific practice [239, 242]. However, more recent work has shown that authenticity is not solely an inherent property of learning settings, but also depends on learners’ individual perceptions of the provided learning environment [71, 243].

**Fig. 10:**
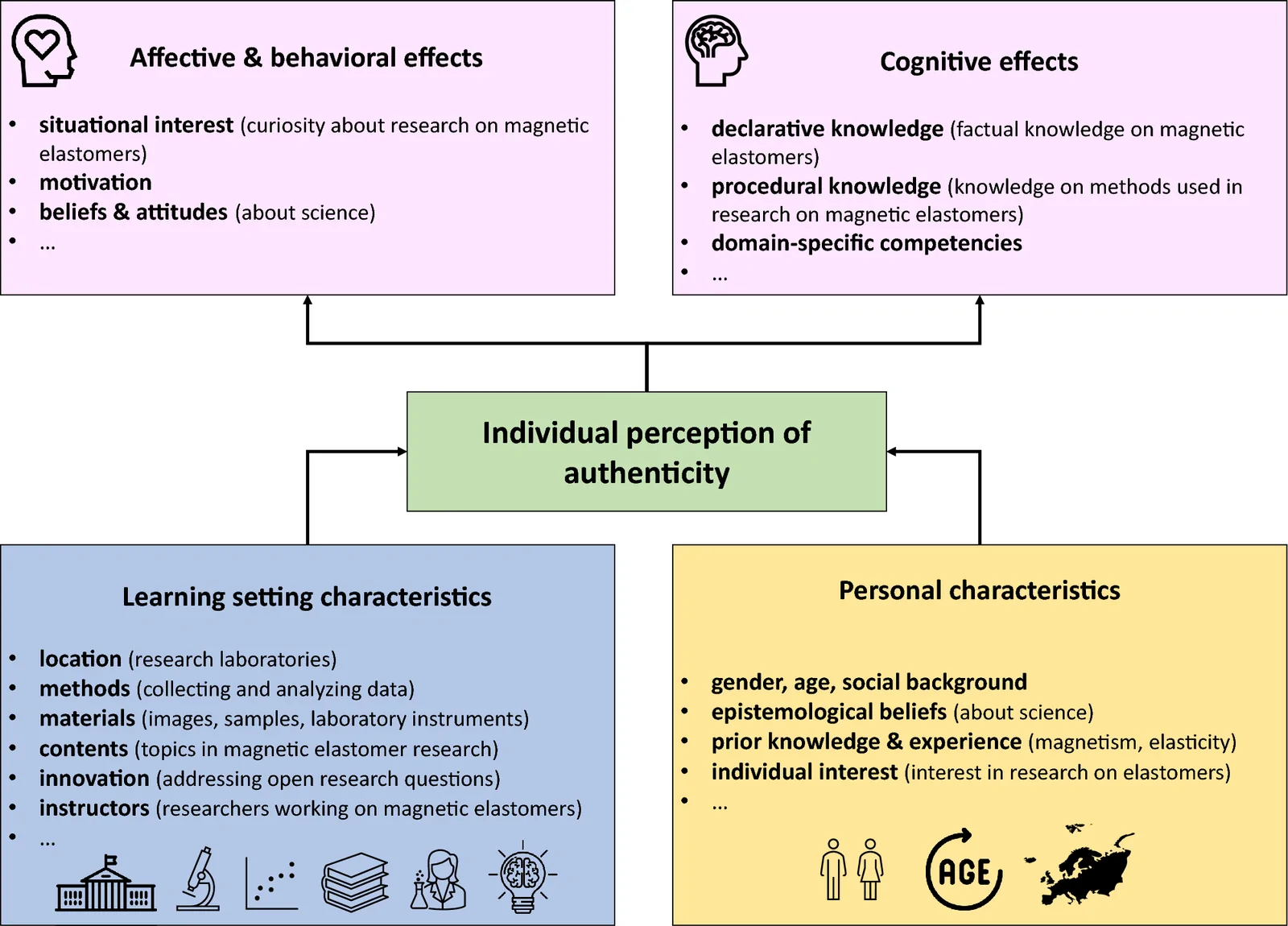
Conceptual model of perceived authenticity, based on the theoretical framework outlined in Ref. [243], adapted from Ref. [248], published under Creative Commons CC BY license. The model illustrates how characteristics of the learning setting and individual learner characteristics interact, resulting in individual perceptions of authenticity and subsequent affective, cognitive, and behavioral outcomes. In the present context, this model is extended by explicitly considering task complexity and cognitive load as additional variables. Examples from research on magnetic elastomers are added in brackets

Authentic learning environments can be characterized by specific design elements such as content, materials, methods, instructors, location, and innovation [243]. These elements (often referred to as “dimensions” of authenticity) may be perceived differently by learners depending on their individual characteristics, prior knowledge, and expectations. As a consequence, the effects of authenticity on affective, cognitive, and behavioral learning outcomes may vary across learners, see Fig. 10. Subsequent theoretical frameworks and empirical studies have supported this multidimensional view of authenticity and have shown that different authenticity dimensions can exert distinct effects on learning outcomes [70, 71, 244].

More specifically, recent research indicates that authentic learning environments are particularly effective in promoting motivational outcomes such as situational interest, relevance perceptions, and engagement [245]. In contrast, findings on cognitive learning outcomes are inconsistent. While some studies report positive effects of authenticity on knowledge acquisition, others find no effects or even negative effects under conditions of high complexity or insufficient instructional support [71, 246]. For example, authenticity related to scientific methods and research practices has been associated with cognitive learning gains under certain instructional conditions [78], whereas instructor-related authenticity primarily appears to affect motivational variables such as situational interest [247]. Material- and location-related authenticity have been shown to influence situational interest and learners’ perceived relevance of the learning content [70, 245], while evidence for effects on knowledge outcomes remains heterogeneous [71].

### Challenges and solutions

One key objective of current research is to explain the conditions under which authenticity supports or hinders motivational and cognitive learning outcomes. However, pursuing this objective is associated with several conceptual and methodological challenges.

A central gap in present research on authenticity is that most empirical work concentrates on outcomes and does not explicitly investigate the underlying pathways. In other words, how does authenticity influence learning processes and why does this influence differ for motivational versus cognitive outcomes?

To address this first challenge, authenticity should not be treated as a single overall factor but as a set of distinct dimensions, see the box “Learning setting characteristics” in Fig. 10, which may contribute differently to learners’ perceived authenticity. In the present context, research on magnetic elastomers offers a suitable case for analyzing the effects of different dimensions of perceived authenticity, because the research topic can be adapted for school-level learning environments. This can be illustrated through the dimension “materials”. For example, school students work with model experiments of macroscopic size, in which magnetizable milli- to centimeter-sized spheres are embedded in a transparent elastic medium. The students directly, visually observe the behavior under the influence of an external magnetic field. Such hands-on model experiments retain their authenticity while being simplified for instructional purposes.

Thus, a multidimensional measurement approach is necessary to analyze authenticity in a differentiated way and to test how specific dimensions of authenticity relate to learning processes and outcomes, for instance, declarative knowledge on magnetic elastomers and interest in the topic.

Accordingly, recent educational research has increasingly developed and validated multidimensional questionnaires that operationalize central dimensions of authenticity and therefore allow dimension-specific analyses of learning processes [244]. However, existing instruments typically capture only selected dimensions of authenticity. In the present subproject, this limitation is addressed explicitly by developing and validating a questionnaire capturing key dimensions of perceived authenticity in science learning contexts. Here, these are *location*, *materials*, *methods*, *content*, *innovation*, and *instructor* [248].

Beyond limitations related to the measurement of authenticity, a second central challenge in research on authentic learning environments concerns the role of complexity. Prior theoretical and empirical research has partially addressed complexity in authentic learning settings, for example, by conceptualizing complexity as a constitutive element of authenticity or by considering learners’ cognitive load during authentic learning tasks [74, 249]. Nevertheless, the interplay between complexity, learners’ cognitive prerequisites, and learning outcomes has, to our knowledge, not yet been examined systematically.

This is surprising given that prominent definitions of authentic learning explicitly emphasize learners’ engagement with complex and ill-structured real-world problems and scientific practices [71, 250]. If, however, the complexity of such problems—for example, in the form of authentic learning tasks—exceeds learners’ cognitive processing capacities, potential motivational and cognitive benefits of authenticity may be diminished or even outweighed by increased cognitive load [251]. Nevertheless, the mechanisms linking learners’ cognitive prerequisites and task complexity in authentic learning contexts to both knowledge acquisition and transfer performance have so far been insufficiently investigated.

**Fig. 11:**
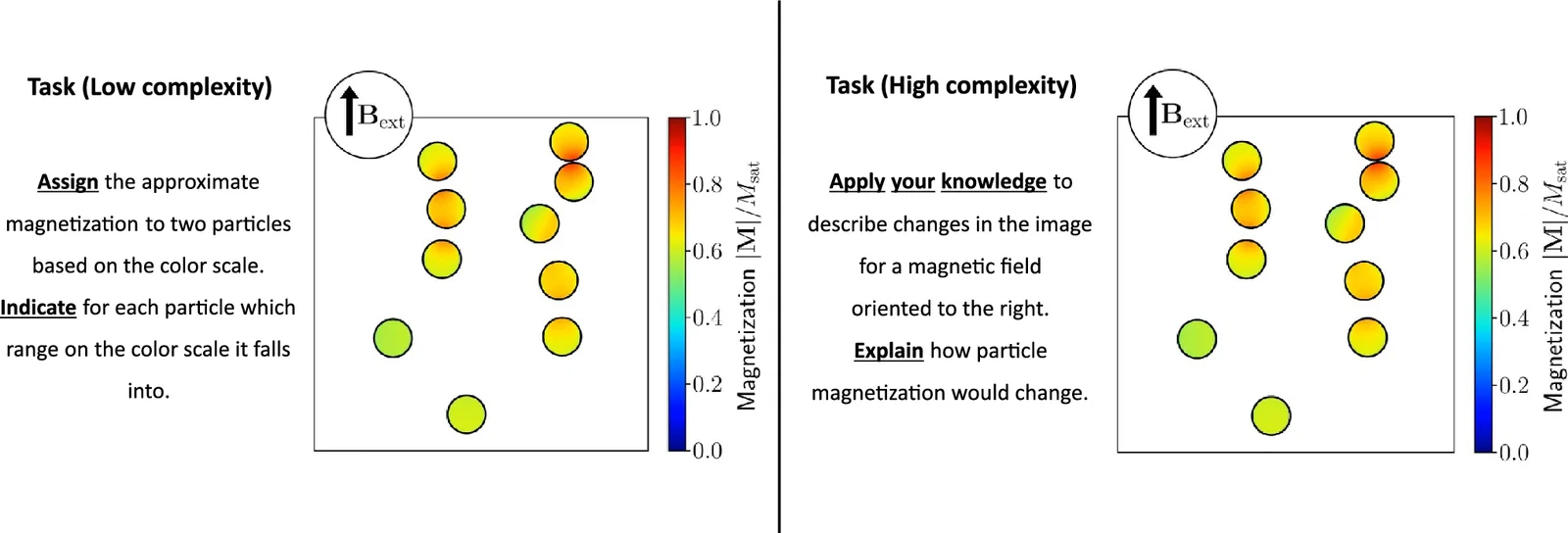
Examples of low- and high-complexity learning tasks based on the same authentic research output, namely, the same figure on the left- and right-hand sides. Here, visualizations of simulated structure formation when generating magnetic elastomers are employed, see Sect. 3. In the low-complexity task (left), students are asked to identify and describe individual aspects of the visualization by assigning approximate (nondimensionalized) magnetization values to two particles using the color scale. In the high-complexity task (right), students are required to mentally transform the situation by changing the direction of the external magnetic field and to explain resulting changes in particle magnetization. Task complexity is varied exclusively through the cognitive demands of the tasks, while the underlying visual representation and materials remain identical

To address this challenge, task complexity needs to be treated as an explicit and independent variable within the aforementioned model of authenticity, see Fig. 10. For this purpose, we manipulate task complexity solely through the cognitive demands of the learning activities that students perform with the same authentic contents [252]. Most importantly, we deliberately refrain from simplifying or redesigning the authentic research output and instead use original visualizations directly from research on magnetic elastomers. Thus, authentic research-based representations form the core learning materials.

An example is given by visualizations of magnetically induced chain formation, see Sect. 3 and Fig. 11. This approach allows us to examine under which instructional conditions authentic research materials can support conceptual understanding and at which point task demands become too high and potentially hinder learning, even if the materials (and other elements of the learning environment) remain authentic. In practice, we implement different levels of task complexity, while keeping the research topic, visualizations, and materials unchanged. Low-complexity tasks are characterized by low element interactivity, meaning that students can focus on individual aspects of the same visualization without the need to relate them to other aspects. Accordingly, these tasks mainly require students to identify and describe relevant elements of the visualization. Higher-complexity tasks involve high element interactivity and require students to simultaneously consider and relate multiple aspects of the same visualization, interpret their relationships, and explain underlying mechanisms and concepts [252, 253].

Beyond these empirical questions, research on magnetic elastomers also offers opportunities for school-level, curriculum-oriented educational applications, as core aspects are not only visually illustrative but can also be made experimentally accessible to school students. For example, simple model experiments with magnetizable macroscopic particles embedded in elastic media can illustrate basic structure-formation processes driven by magnetic interactions. Comparatively simple magnet configurations, such as linear Halbach arrays, see Sect. 2, can provide a bridge to established topics in school physics and be used to deepen students’ understanding of the superposition of magnetic fields and of field homogeneity [254]. In addition, students can be introduced to scientific practices by describing magnetic configurations, varying parameters such as magnet arrangements, comparing model-based predictions or simulations with experimental observations, and drawing evidence-based conclusions from their measurements.

### Conclusions and outlook

Research on authentic learning environments has progressed beyond the simple assumption that more authenticity is always better. Current evidence suggests that authenticity consists of different components and that its effects depend on the learning context and the learners themselves. In particular, many studies report strong motivational effects, for example, interest and perceived relevance. Yet, results on conceptual understanding and knowledge gains are less consistent. This indicates that authenticity alone does not automatically lead to better learning.

Thus, we take a more differentiated approach. Rather than treating authenticity as a single overall factor, we distinguish between different authenticity dimensions. We investigate which of them are relevant for particular instructional goals. This allows us to better understand the conditions under which authenticity supports cognitive learning, and under which it may not.

A central direction for future research is to integrate task complexity more explicitly. Authentic scientific contexts are often inherently complex. Therefore, complexity should be treated as a central variable rather than an unavoidable side effect. By systematically varying the cognitive demands of learning tasks, while keeping authentic materials unchanged, future studies can examine how authenticity and complexity jointly influence both motivational and cognitive outcomes to identify an “optimal range” of task demands.

In this regard, the present study on authenticity in the context of structured magnetic elastomers can illustrate how authentic research materials can be retained while the complexity of the learning tasks is systematically adapted to learners’ prior knowledge and abilities. The expected contribution therefore lies not only in the development of illustrative learning materials, but also in deriving empirically grounded recommendations for adapting task complexity when authentic research is transformed into school-level learning environments.

### Acknowledgments

We acknowledge support by the German Research Foundation DFG (Deutsche Forschungsgemeinschaft) through Research Unit FOR 5599 on structured magnetic elastomers, project nos. 511114185 and 535548756.

## Overall conclusions and perspective

Henning Reinken, Karl A. Kalina, Deniz C. Senel, Heinrich T. Roth, Nils Magin, Konstantin Gerstenberger, Markus Kästner, Stefan Odenbach, Bianca Watzka, Günter K. Auernhammer, Andreas M. Menzel, *Otto-von-Guericke-Universität Magdeburg, Magdeburg, Germany, Technische Universität Dresden, Dresden, Germany, RWTH Aachen University, Aachen, Germany, Leibniz-Institut für Polymerforschung Dresden e. V., Dresden, Germany*

In summary, we have overviewed how a concerted action of various disciplines can serve to develop a deeper understanding of the fabrication process and potential in material properties of structured magnetic elastomers. In the presented case, this strategy combines experimental and computational approaches to reveal the background of structure formation on the microscopic particle scale, see Sects. 2 and 3, respectively. Likewise, experimental and computational approaches serve to link such microscopic properties to overall material behavior, see Sects. 4 and 5, respectively. Finally, educational sciences explore ways of transmitting corresponding knowledge to others, starting from the level of school education, see Sect. 6, together with fascination of STEM subjects in general.

With respect to important next steps, we first note extensions to three-dimensional studies concerning the microscopic investigations of structure formation on the particle scale. For this purpose, an upgraded three-dimensional Halbach array enables adjustable, highly homogeneous magnetic fields in three dimensions. Moreover, a systematic combination of template and capillary-assisted particle placement methods can achieve well-defined two- and three-dimensional particle arrangements. One step further, individual particle dynamics can be tracked through optical velocimetric methods in three dimensions at high spatial and temporal resolution, particularly while the crosslinking process of the surrounding elastomer takes place during structure formation. Related to this point, on the theoretical side, in view of particle-based simulations, an important future milestone is to include viscoelastic and elastic effects [255–259]. Such extensions enable to investigate particle aggregation during changing material properties, for example, during the chemical crosslinking process and formation of the permanent elastic confinement. Specifically, this includes extensions beyond the linear regime. On this basis, significant restructuring in the final, elastic material can likewise be analyzed on the particle scale.

More specific next steps in structural explorations of whole materials consist of turning from “permanently unfinished states” to “frozen states” in structural X-ray tomographic analysis. In such “frozen states”, the solidification of the surrounding polymer is reversible. Through an external control mechanism, the elastomeric solid can be rendered fluid again, so that further particle rearrangement becomes possible, before resolidification for further X-ray analysis is initiated. A major future challenge lies with identifying and establishing an appropriate corresponding system. Besides, integrating a large electromagnet within a novel tomography set-up enables to implement time-controlled fields during the structuring process. In combination, a sequence of “frozen states” under repeated field application, would allow for a more continuous study of structure formation persistently on only one sample of material continuously present in the tomography set-up. On the computational side, in finite-element simulations, major limitations at the moment arise from instabilities when particles approach each other towards mutual contact. Therefore, stable finite-element formulations for this scenario still represent a challenging future task.

Concerning the connection of microscopic structural properties to overall material behavior, an important next step is the efficient generation of statistical volume elements from microscopic experimental and theoretical analysis and from X-ray microcomputed tomography data as an input to scale-bridging simulations. Moreover, such microstructural elements should be generated for different ways of conducting the process of structure formation, based on the information from the different approaches. Resulting evaluations can be used to calibrate a parameterized physics-augmented neural network, which, as an ultimate goal, does not only link microscopic properties in finalized materials to macroscopic material behavior, but also includes the process of structure formation. The resulting model can then be used to invert process–structure–property relations to identify ways of process control of material preparation to achieve optimized overall material behavior.

From the educational perspective, a central step is to investigate how authentic learning environments in the context of structured magnetic elastomers can be designed to support students’ cognitive and affective learning processes. To this end, developing school-appropriate experiments and learning materials of varying complexity serves to examine how perceived authenticity interacts with task complexity and cognitive load. They help to clarify under which conditions learning outcomes such as conceptual understanding and interest in the topic are effectively supported. In this regard, magnetic elastomers and their structuring process are useful example systems to convey basic concepts such as elasticity and magnetism, for example, in macroscopic hands-on experiments. Moreover, our work can contribute to further systematic investigations of the interplay between authenticity, complexity, and cognitive load. Structured magnetic elastomers provide a beneficial context for examining how these variables interact and how this interplay relates to cognitive and affective outcomes in science learning. Future studies may build on these results to examine under which conditions authentic learning environments support cognitive and affective outcomes in different STEM contexts. At the same time, they serve to stimulate and convey fascination for STEM subjects.

In view of the increasing significance, for example, of automated schemes and AI tools, establishing high-quality databases is becoming of central importance. Following the FAIR principles of findability, accessibility, interoperability, and reusability [260], databases for structured magnetic elastomers should be grounded in systematic research data management. Their value does not only lie in collecting measurement or simulation files, but especially in the documentation that makes these data interpretable, reproducible, and reusable. As a link to real-world workflow, electronic laboratory notebooks allow to document experimental and computational advances in a structured and searchable form, to connect datasets directly to protocols and analysis steps, and to record decisions during data acquisition and interpretation transparently. Datasets from fabrication, observations of structure formation, rheological benchmark measurements, tomography, and simulations should be connected to contextual information, that is, metadata, such as measurement settings, preprocessing decisions, simulation parameters, software versions, and scripts of analysis. Ideally, this should be realized through standardized metadata schemes, open and machine-readable formats, persistent identifiers, and version control. The notebooks should provide links to the actual data repositories. Through this connection, a basis for later automated readout and reusage of the data, or also automatization of the whole process of data acquisition, metadata documentation, and data storage, is laid. Standardization remains a major challenge in databases established from interdisciplinary work.

The strategies overviewed in this article serve to build foundations and enhance fundamental understanding. They support the way towards actual use and application of structured magnetic elastomers as a tailored subclass of magnetic elastomers in general. In educational settings, such use of structured magnetic elastomers has already become immediate. They can serve as authentic examples of materials whose properties can be controlled by external magnetic fields. Moreover, they appeal by their interdisciplinary combination of aspects of research in physics, chemistry, and materials science. Individual aspects of this current research, such as polymer properties, magnetic interactions, or the generation of strong, uniform magnetic fields, can be connected to established curricular contents in schools. For example, Halbach arrays make the concepts of homogeneity and of superposition of magnetic fields directly accessible in experiments [254].

Concerning the use of structured magnetic elastomers with optimized particle arrangements in actual, practical applications, there are manifold examples. At this point, we can only provide a brief insight. We mention their promising potential in view of adaptive systems. Their enhanced magnetomechanical response enables applications as tunable valves [105, 261, 262], dynamic vibration absorbers, and vibration isolators [263–265]. Furthermore, advances in controlled manufacturing of anisotropic magnetic composites support the development of soft robotic systems and reconfigurable soft actuators with programmable shape changes [20, 266–273]. Additional areas of application include minimally invasive medical devices requiring precise, remotely controlled actuation [274], also stretchable inductors [275], as well as magnetic sensing technologies based on tailored particle architectures [276, 277]. Other sensing applications concern acceleration sensors [278, 279] and force sensors [280].

For such technological applications, durability is an important aspect that requires more in-depth investigation in the future [281]. Generally, elastomeric components are subject to degradation caused by a variety of factors, including sunlight and UV radiation, thermal and pressure-related factors, and chemical reactions [282–286]. For example, clear and quantifiable degradation was demonstrated by ninety days of exposure to environmental factors in a tropical climate [284]. To counteract these effects, protection against environmental influences to the greatest possible extent is crucial.

Similarly, service lifetime can be reduced by the presence of the magnetic components. Oxidation of the polymeric material can be enhanced by larger concentrations of magnetic particles [287]. Additionally, the oxidation of the particles themselves can substantially alter the overall material properties, for example, implying a reduced magnetorheological effect. In such instances, it is required that the metal particles be treated with an antioxidation coating [288].

Moreover, repeated application of magnetic fields can lead to an irreversible change in the microstructure and thus affect the overall response. Localized internal fractures of the elastomeric component may occur during repeated cycling, and potential bonds between the particles and the confining elastomer may be destroyed, resulting in behavior that is significantly altered over time [289, 290]. Therefore, in the future, enhancing the durability of the materials during practical use has the potential to develop into an important subfield of corresponding materials research.

In summary, we may conclude that ongoing research on structured magnetic elastomers will enhance not only our fundamental understanding but also their potential in view of actual applications. It is important to understand and control underlying processes of structure formation, material preparation, and their links to overall properties to be able to achieve the most beneficial outcome in the performance of these materials. Disseminating corresponding results, stimulating research on actual applications, and encouraging investigations on increased durability of the materials are important future aspects from a practical perspective. We envision that our work lays a solid basis in the framework of fundamental research to support such broad exploration of the materials.

## Data Availability

Due to the nature of this roadmap article, there is no immediate research data associated with it that is not published or shall not be published in the context of other publications. The corresponding original research articles are cited. Figures that have been reproduced are marked correspondingly and are amended by appropriate license information in the figure captions.
